# Coordinating interleukin-2 encoding circRNA with immunomodulatory lipid nanoparticles to potentiate cancer immunotherapy

**DOI:** 10.1126/sciadv.adn7256

**Published:** 2025-02-26

**Authors:** Kai Yang, Bing Bai, Xiaomei Li, Wei Rou, Cheng Huang, Meixin Lu, Xueyan Zhang, Chunbo Dong, Shaolong Qi, Zhida Liu, Guocan Yu

**Affiliations:** ^1^ Ministry of Education Key Laboratory of Bioorganic Phosphorus Chemistry & Chemical Biology, Department of Chemistry, Tsinghua University, Beijing 100084, P. R. China.; ^2^ Shanxi Academy of Advanced Research and Innovation, Taiyuan 030032, P. R. China.; ^3^ MOE Key Laboratory of Coal Environmental Pathogenicity and Prevention, Shanxi Medical University, Taiyuan 030001, P. R. China.; ^4^ Engineering Research Center of Advanced Rare Earth Materials, Department of Chemistry, Tsinghua University, Beijing, 100084 P. R. China.

## Abstract

Interleukin-2 (IL-2) is a cytokine vital for CD8^+^ T cell activation and proliferation, holding great potential for cancer immunotherapy. Nevertheless, inherent shortcomings of short half-life, activation of regulatory T (T_reg_) cells, and systemic toxicity limit its application. To tackle these, a circular RNA (cRNA)–based IL-2 therapy using immunomodulatory lipid nanoparticles [ursodeoxycholic acid lipid nanoparticles (ULNPs)] and sustained-release hydrogel was developed. Fusing fragment crystallizable (Fc) region into IL-2 and encoding this fusion protein IL-2-Fc (IL-2F) in cRNA (cRNA^IL-2F^) greatly extend the half-life. ULNPs containing ursodeoxycholic acid, a transforming growth factor-β1 inhibitor, suppress the function of T_reg_ cells. Consequently, the ULNPs-cRNA^IL-2F^ formulation promotes CD8^+^ T cells and suppresses T_reg_ cells, increasing the CD8^+^/T_reg_ ratio for effective immunotherapy. Furthermore, a locally administrated hydrogel loading with ULNPs-cRNA^IL-2F^ sustains the release, enhancing efficacy and reducing toxicity. This innovative approach achieves remarkable tumor inhibition in both melanoma and orthotopic glioma models with or without surgery, offering a promising future for cancer immunotherapy.

## Teaser

A circRNA–based IL-2 therapy features prolonged IL-2 half-life, low T_reg_ activation, and high safety for cancer immunotherapy.

## INTRODUCTION

Interleukin-2 (IL-2) plays a crucial role in tumor immunotherapy, stimulating the proliferation of CD8^+^ T cells and enhancing the body’s immune response against cancer cells (*1*–*4*). However, the inherent limitations of IL-2 greatly restrict its clinical applications (*5*, *6*). Specifically, the short half-life, toxicity, and limited tumor penetration hinder the elicitation of an efficient and durable antitumor immune response (*7*). Notably, IL-2 not only specifically activates CD8^+^ T cells, but also promotes the generation and function of immunosuppressive regulatory T (T_reg_) cells, which dampens the antitumor efficacy of IL-2 (*8*). Moreover, intravenous administration results in extensive exposure of IL-2 to the circulatory system. While this enhances immune cell recruitment, it also inevitably leads to severe systemic toxicity and potential fatality (*9*). Therefore, it is crucial to achieve sustained presence of IL-2 and an increased CD8^+^ T/T_reg_ ratio at tumor sites, simultaneously reducing its systemic exposure and mitigating adverse effects.

By using mRNA to exploit the body as a factory for therapeutic proteins, enabling sustained in vivo protein production with minimized peak plasma concentration (*10*), mRNA therapeutics has emerged as a potent approach for cancer treatment. However, the low stability and immunogenicity of mRNA greatly impair their clinical applications as a tool for protein replacement therapy that requires multiple injection with extremely high dosage. Compared to linear mRNA, circular RNA (cRNA) with minimized immunogenicity features a covalently closed loop structure and lacks a 5′ cap and 3′ poly-A tails, rendering it more resistant to exonuclease and capable of stable cellular existence (*11*, *12*), which prolongs the expression of therapeutic proteins and extends their persistence in the body (*13*). Besides, Fc fusion represents an additional approach to enhance protein stability and increase in vivo half-life (*14*, *15*). Therefore, we hypothesize that the employment of IL-2F fusion protein encoding cRNA (cRNA^IL-2F^) for in situ production of IL-2F at the tumor site may overcome the limitations associated with conventional IL-2 therapy.

Lipid nanoparticles (LNPs) serve as efficient vehicles for the delivery of nucleic acids, but meaningful theranostic properties that can be ingeniously incorporated into LNPs have yet to be explored (*16*, *17*). Ursodeoxycholic acid (UDCA), an approved drug for the treatment of cholestasis-related diseases, has recently been found to inhibit transforming growth factor–β1 (TGF-β1) and eventually suppress the function of T_reg_ cells (*18*, *19*). Given that UDCA is a hydrophobic steroid with a chemical structure similar to cholesterol, one component of LNPs, we hypothesize that UDCA can substitute cholesterol to assemble LNPs through hydrophobic interactions, affording immunomodulatory UDCA-LNPs (ULNPs). It is expected to exert the inhibitory effect of UDCA on T_reg_ cells to improve antitumor efficacy by counteracting the activation of T_reg_ cells by IL-2. In addition, the systematic biodistribution of IL-2 can cause nonnegligible side effects, which can be mitigated by local administration. Of note, hydrogel with a porous structure is expected to efficiently load ULNPs and confine the cargoes within the administration site to evade adverse effects, in which the payloads are released in a controlled manner as hydrogel gradually degrades (*20*–*22*). With the incorporation of hydrogel and intratumoral administration, we assume that the ULNPs-cRNA^IL-2F^ would be confined to the administration site, thus establishing a localized high-concentration drug release area while minimizing systemic exposure.

**Fig. 1.**
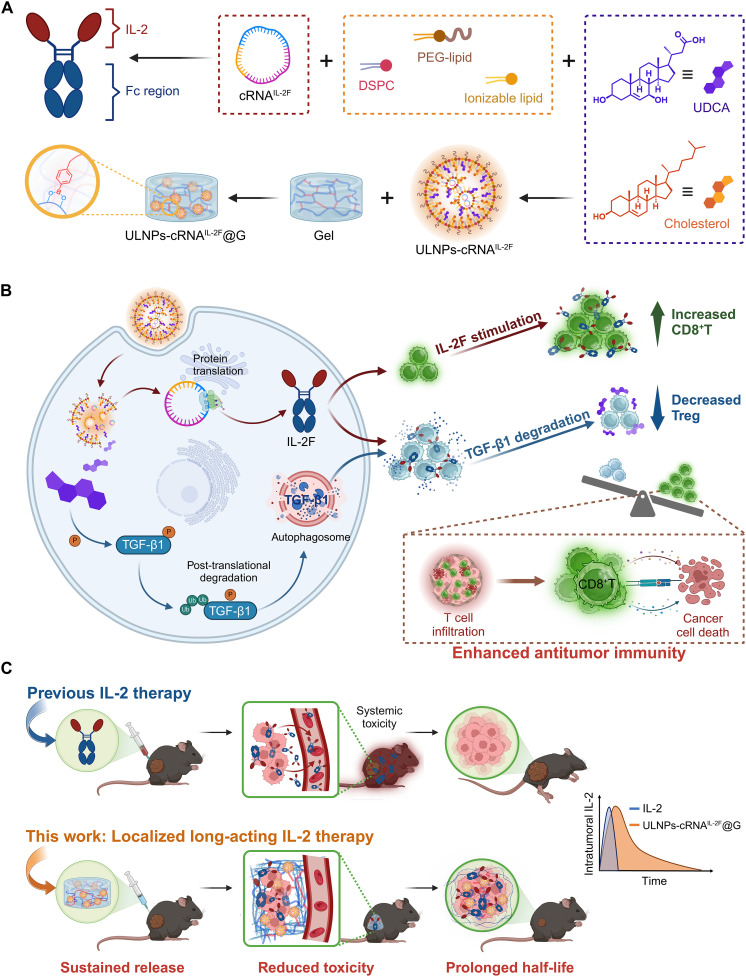
Schematic illustration of the design of ULNPs-cRNA^IL-2F^@G. (**A**) The preparation of ULNPs-cRNA^IL-2F^@G. (**B**) The antitumor mechanism. ULNPs-cRNA^IL-2F^@G reconstructed the immune cell composition within the tumor microenvironment by translating cRNA^IL-2F^ and facilitating the posttranslational degradation of TGF-β1. (**C**) The therapeutic advantages of ULNPs-cRNA^IL-2F^@G. Compared to IL-2, the formulation of ULNPs-cRNA^IL-2F^@G enhanced the intratumoral level of IL-2F and reduced its systemic toxicity.

Herein, we report a sophisticated cRNA-based IL-2 therapy by incorporating immunomodulatory LNPs and hydrogel for potent cancer immunotherapy with alleviated side effects (Fig. 1). An immunomodulatory LNP containing UDCA is developed to encapsulate cRNA^IL-2F^, giving the nanoformulation ULNPs-cRNA^IL-2F^, which is capable of promoting the proliferation and activation of CD8^+^ T cells while inhibiting the generation and function of T_reg_ cells to maximize the antitumor efficacy. To further prolong the expression of IL-2F and alleviate side effects, ULNPs-cRNA^IL-2F^ is loaded in a degradable hydrogel and administrated intratumorally. The incorporation of hydrogel substantially extends the duration and magnitude of IL-2F expression, while also localizing IL-2F within the tumor site, thereby inducing potent T cell immunity at the tumor site and minimizing systemic dissemination. This innovative therapy achieves remarkable tumor inhibition in both melanoma and orthotopic glioblastoma (GBM) models and is also applicable in clinically relevant models for preventing postsurgical tumor recurrence. This innovative IL-2 therapy achieves remarkable tumor suppression in both heterotopic melanoma and orthotopic GBM models, and is also applicable in preventing postsurgical tumor recurrence. Moreover, the enhanced safety of this therapy is demonstrated by lower levels of IL-2, alanine aminotransferase (ALT), TNF-α, and interferon-γ (IFN-γ) in the serum compared to mice treated with commercialized LNP-encapsulated cRNA^IL-2F^, highlighting its superior therapeutic profile.

## RESULTS

### Preparation and characterization of the ULNPs-gel system

**Fig. 2.**
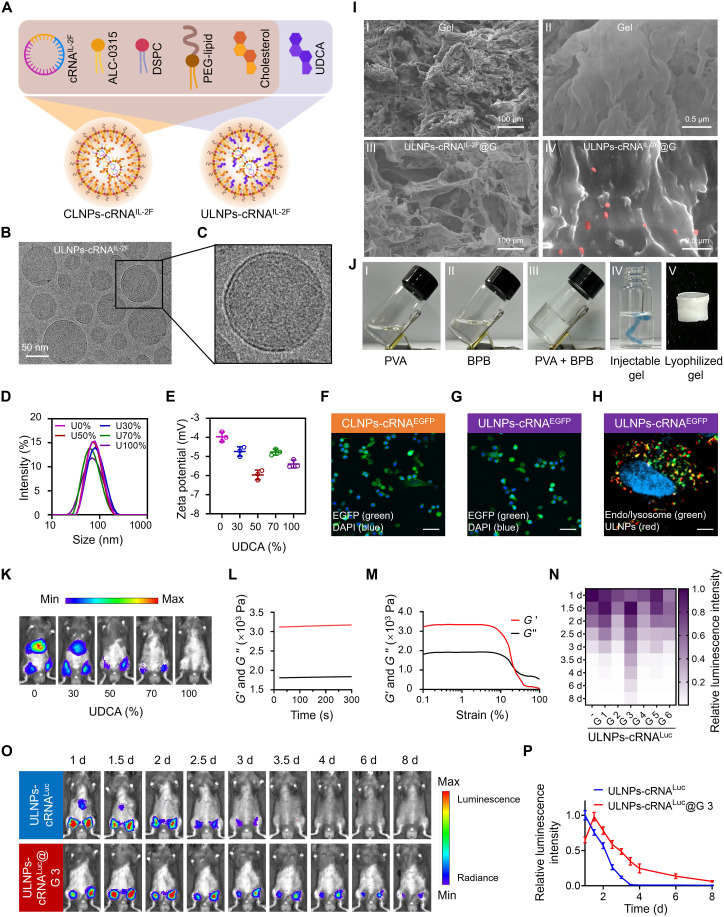
Design and characterization of the ULNPs-gel system. (**A**) Schematic illustration of CLNPs-cRNA^IL-2F^ and ULNPs-cRNA^IL-2F^. (**B** and **C**) Cryo-TEM image of ULNPs-cRNA^IL-2F^. DLS results (**D**) and zeta potential (**E**) of ULNPs-cRNA^IL-2F^ containing different proportions of UDCA. (**F** and **G**) CLSM images of B16F10 cells incubated with CLNPs-cRNA^EGFP^ or ULNPs-cRNA^EGFP^ for 24 hours (scale bar, 50 μm). (**H**) CLSM images of B16F10 cells treated with Rhodamine 6G–labeled ULNPs-cRNA^IL-2F^ for 8 hours. DAPI and Lysotracker Green were used to stain nuclei and endo/lysosomes, respectively. Scale bar, 5 μm. (**I**) SEM images of Gel (I and II) and ULNPs-cRNA^IL-2F^@G (III and IV). ULNPs-cRNA^IL-2F^ were shown in pseudo-colored red. (**J**) Photographs of PVA (I), BPB (II), and mixture of PVA and BPB (III). The prepared hydrogel was simply mixed with Evans blue and injected into the water through a syringe to characterize the injectable gel (IV). The prepared hydrogel was freeze dried to form the lyophilized gel (V). (**K**) Bioluminescence imaging of the mice injected with ULNPs-cRNA^Luc^ containing different proportions of UDCA. (**L**) Variation in the moduli of the gel over time. (**M**) Amplitude sweep of *G*′ and *G*″. (**N**) The heatmap of relative luminescence intensity at the injected sites for the mice injected with ULNPs-cRNA^Luc^ and ULNPs-cRNA^Luc^@G. d, days. (**O**) Bioluminescence images of the mice injected with ULNPs-cRNA^Luc^ and ULNPs-cRNA^Luc^@G 3 at different times postinjection. (**P**) Quantitative results of relative luminescence intensity from (O).

LNPs consist of four components including ionizable lipid, helper lipid, lipid-polyethylene glycol, and cholesterol, monotonously serving as a delivery vehicle for nucleic acids. The chemical similarity between UDCA and cholesterol inspired us to explore a strategy that partially substitutes UDCA for cholesterol in LNPs to develop immunomodulatory ULNPs without diminishing their delivery performances. On the basis of a commercially available LNP (CLNP) formulation, the cholesterol was replaced by UDCA at ratios of 30, 50, 70, and 100% (Fig. 2A). To assess the impact of UDCA at different ratios on LNPs, we evaluated the hydrodynamic size and zeta potential of the obtained ULNPs in phosphate-buffered saline (PBS, which showed negligible differences in the average diameter and surface charge (Fig. 2, D and E), indicating that the replacement of cholesterol by UDCA showed no disturbance of the self-assembly process with other lipids. To evaluate the translation efficiency of ULNPs, firefly luciferase-encoding cRNA (cRNA^Luc^) was encapsulated by ULNPs and intramuscularly injected into the bilateral inner thighs of mice. The results showed that ULNPs with 30% replacement of cholesterol was the optimum replacement ratio that ensured sufficient drug loading without attenuating the excellent translation efficiency of cRNA^Luc^ in vivo (Fig. 2K). Given the complexity of physiological environment, ULNPs were incubated in 10% fetal bovine serum (FBS) to disclose the relationship between UDCA ratio and translation efficiency. After incubation in PBS containing 10% of FBS for 24 hours, the average size of ULNPs gradually increased, especially for the ones with a high UDCA ratio (fig. S1). Because cholesterol has been proven to contribute to maintaining the stability of LNPs (*23*), we supposed the high–UDCA ratio formulations were unstable in the physiological environment, which might impair the translation efficiency. Therefore, ULNPs with 30% cholesterol replacement by UDCA were used for the following studies. Morphological analysis by cryo–transmission electron microscopy (cryo-TEM) presented that ULNPs had a spherical structure and uniform distribution, with an average size of 80 nm (Fig. 2, B and C). Next, we examined the intracellular uptake and trafficking of ULNPs in B16F10 cells by confocal laser scanning microscopy (CLSM). ULNPs encapsulating the enhanced green fluorescent protein–encoding cRNA (cRNA^EGFP^) were incubated with B16F10 cells for 24 hours. CLSM images showed that the EGFP-positive cells incubated with ULNPs-cRNA^EGFP^ were comparable to those of CLNPs-cRNA^EGFP^ (Figs. 2F and 1G and fig. S2). We further evaluated the endo/lysosomal escape capability of ULNPs, which demonstrated that most of the red signal from Rhodamine 6G–labeled ULNPs was not colocalized with the green signal from lyso/endosomes after 8 hours of incubation, indicating that ULNPs effectively escaped and translocated into the cytoplasm (Fig. 2H).

To realize the localized production and release of proteins translated by the loaded cRNA, we fabricated a hydrogel to load ULNP-cRNA formulations. Considering the destabilizing impact of cationic and anionic hydrogels on LNP stability via electrostatic interactions, and the potential reactivity of amino and carboxyl groups in polysaccharide-based hydrogels with the phospholipids in LNPs, we developed a neutral hydrogel with a dynamic and degradable network based on borate ester bonds. A phenylboronic acid functionalized polyethylene glycol (BPB) was synthesized to form the cross-linked network with poly(vinyl alcohol) (PVA) via the dynamic bond between hydroxy groups on PVA and phenylboronic acids on BPB (figs. S3 to S5). As shown in Fig. 2J, a solution-hydrogel transition occurred when PVA and BPB were mixed, indicating the formation of a cross-linked network. Given that the mechanical properties of the hydrogel could affect the release and translation of CLNPs-cRNA (*24*), the strategy that using BPB as a cross-linker allowed us to facilely control the cross-linked density by adjusting the PVA-to-BPB ratio, thereby regulating release kinetics and optimizing in vivo translation efficiency. As shown in Fig. 2N and figs. S7 and S8, ULNPs-cRNA^Luc^ or ULNPs-cRNA^Luc^@Gel (ULNPs-cRNA^Luc^@G) with different Young’s moduli were subcutaneously injected into the bilateral inguinal of mice to evaluate the sustained release performance. Encouragingly, in vivo expression in the Gel 3 group lasted up to 8 days; however, the bioluminescence signal rapidly declined for ULNPs-cRNA^Luc^. These results indicated that Gel 3 effectively preserved the stability of its loaded ULNPs-cRNA^Luc^ and facilitated sustained release. Besides, bioluminescence signals were detected at both the injection site and abdomen for the mice injected with ULNPs-cRNA^Luc^, whereas the incorporation of hydrogel successfully confined the ULNPs-cRNA^Luc^ to the administration site and avoided the unwanted accumulations in other organs (Fig. 2O), which was extremely important to minimize systemic exposure of the therapeutic cRNA and translated proteins. The mechanical properties of Gel 3 were assessed by a rheological study, which indicated that the Young’s modulus (*G*′) was much higher than the elastic modulus (*G*″), a convincing evidence for the formation of the cross-linked network (Fig. 2, L and M). Meanwhile, scanning electron microscopy (SEM) also showed a highly cross-linked and porous structure of Gel 3 (Fig. 2I). After encapsulating with ULNPs, the enlarged SEM showed that ULNPs were successfully embedded in the hydrogel (Fig. 2I).

### In vitro synthesis and evaluation of cRNA^IL-2F^

**Fig. 3.**
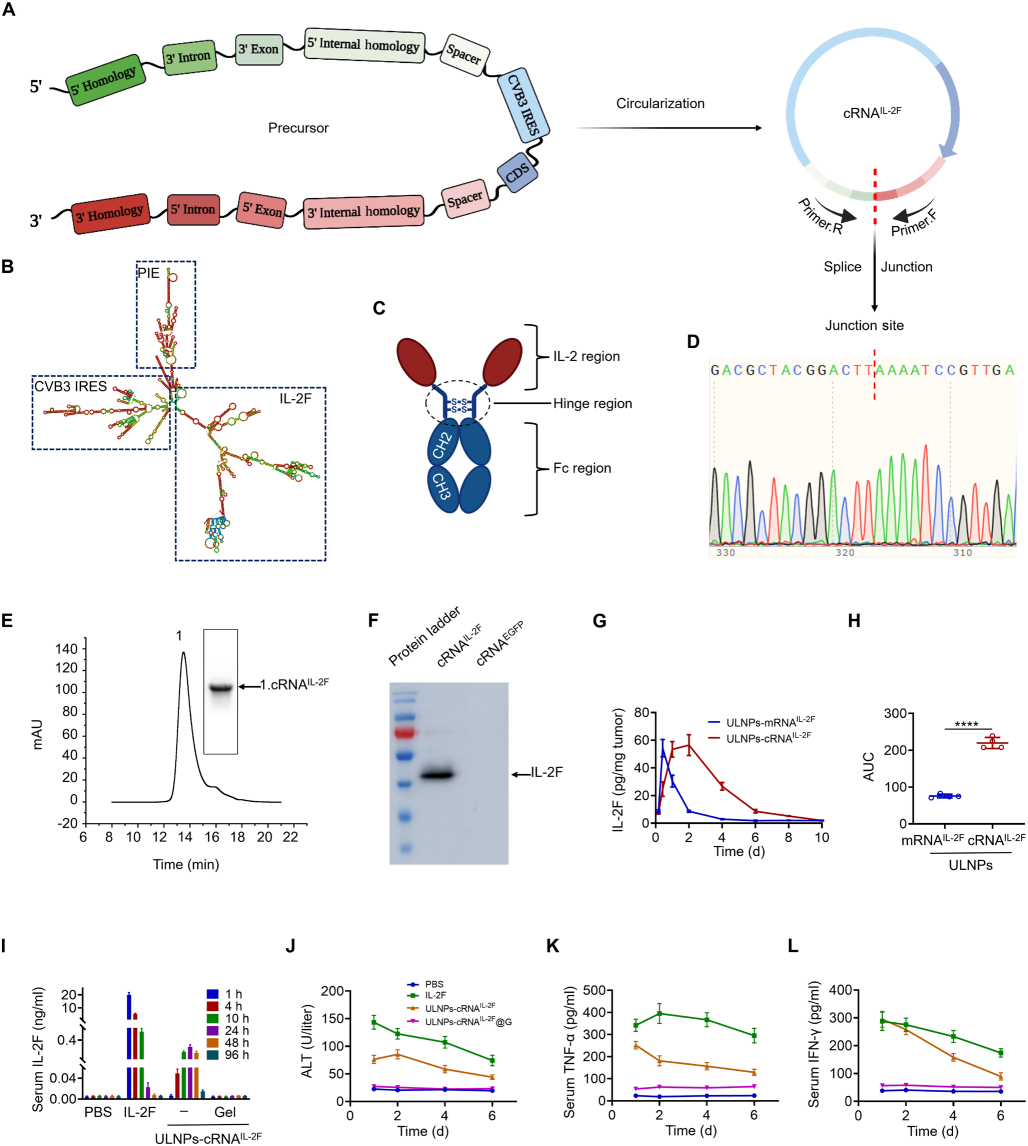
Synthesis and characterization of the cRNA^IL-2F^. (**A**) The design of cRNA^IL-2F^ sequence. (**B**) RNAFold predictions of the precursor RNA secondary structure. (**C**) Schematic diagram of the IL-2F construct. (**D**) Sanger sequencing result of the RT-PCR cRNA^IL-2F^. The junction site is marked by the red dotted lines. (**E**) HPLC result of cRNA^IL-2F^ from splicing reactions after purification. Inset is the agarose gel of collected fractions. (**F**) Immunoblot analysis of IL-2F in the culture supernatant of HEK293T cells transfected with cRNA^IL-2F^ using the Lipofectamine MessengerMAX transfection reagent. (**G**) ELISA analysis of IL-2F in the tumor site. (**H**) AUC of IL-2F content from (G). ELISA analysis of serum IL-2F (**I**), TNF-α (**K**), and IFN-γ (**L**) at different time points after different treatments. (**J**) Biochemical analysis of serum ALT level. Data represent means ± SD. Statistical difference was calculated using Student’s *t* test (****P < 0.0001). d, days.

In this study, the permuted intron-exon (PIE) system was applied to synthesize cRNA^IL-2F^. As shown in Fig. 3A, a linear RNA template was designed to include the IL-2 coding sequence, Fc region sequence, and necessary linkers. A cap-independent coxsackievirus B3-derived internal ribosome entry site (CVB3-IRES) was inserted before the coding sequence for translation initiation. To improve the circularization efficiency of self-splicing precursor RNA, homology arms and spacer sequences were added to the precursor molecule. The relevant precursor RNA secondary structure is shown in Fig. 3B. Sanger sequencing confirmed the sequence around the junction site and validated the circularization of cRNA^IL-2F^ (Fig. 3D). The final product of the splicing reaction was purified by high-performance liquid chromatography (HPLC), showing a single sharp peak corresponding to the cRNA^IL-2F^ product, confirming the effective purification (Fig. 3E). Western blotting analysis revealed a distinct band at the expected molecular weight range consistent with the fusion protein size, confirming the successful expression of the IL-2F fusion protein (Fig. 3F).

The closed-loop structure of cRNA^IL-2F^ makes it resistant to exonuclease degradation, enabling it to persist longer in the cell than linear mRNA^IL-2F^. This enhanced stability offers more opportunities for cRNA^IL-2F^ to serve as a template for IL-2F translation. To assess the expression capability of cRNA^IL-2F^, B16F10-bearing mice were intratumorally injected with either ULNPs-mRNA^IL-2F^ or ULNPs-cRNA^IL-2F^. Encouragingly, the result demonstrated that in the ULNPs-cRNA^IL-2F^ group, the intratumoral level of IL-2F gradually peaked within 2 days and maintained elevated expression for up to 10 days. In contrast, the intratumoral level of IL-2F rapidly peaked within 10 hours and then rapidly decreased over the next 4 days for the mice administrated with ULNPs-mRNA^IL-2F^ (Fig. 3G). The area under the curve (AUC) of IL-2F level in the ULNPs-cRNA^IL-2F^ group is 2.9 times that of ULNPs-mRNA^IL-2F^ (Fig. 3H), confirming that cRNA^IL-2F^ had a prolonged expression capability and elevated the intratumoral production of therapeutic IL-2F.

To evaluate the systemic toxicity, the mice were intratumorally injected with PBS, IL-2F (2 μg per mouse), ULNPs-cRNA^IL-2F^ (2 μg cRNA^IL-2F^ per mouse), and ULNPs-cRNA^IL-2F^@G (2 μg cRNA^IL-2F^ per mouse). Figure 3I and fig. S10 show that the IL-2F levels in serum of the mice treated with IL-2F and ULNPs-cRNA^IL-2F^ were 117.0- and 27.7-fold higher than that of PBS. In sharp contrast, mice treated with ULNPs-cRNA^IL-2F^@G showed a similar serum IL-2F level to the PBS group, which verified that the produced IL-2F merely penetrated the bloodstream. Noteworthily, the intratumoral level of IL-2F in the ULNPs-cRNA^IL-2F^@G group was 4.3 and 2.3 times higher than those in the IL-2F and ULNPs-cRNA^IL-2F^ group, respectively (Fig. 5E). In addition, serum levels of TNF-α and IFN-γ that mediated inflammation and immune responses were found to be much lower in the mice treated with ULNPs-cRNA^IL-2F^@G than those from the IL-2F and ULNPs-cRNA^IL-2F^ groups, suggesting the reduced systemic toxicity (Fig. 3, K and L). Consistent with the changes in cytokines, ALT levels (24 hours after administration) were greatly elevated in the IL-2F and ULNPs-cRNA^IL-2F^ groups compared to PBS (6.4 and 3.4 times, respectively), whereas the ALT level in the ULNPs-cRNA^IL-2F^@G group remained comparable to PBS (Fig. 3J). These data indicated that the application of hydrogel confined the production and release of IL-2F to the administration site, thus avoiding the systemic exposure of IL-2F.

### The mechanism of TGF-β1 degradation induced by UDCA

**Fig. 4.**
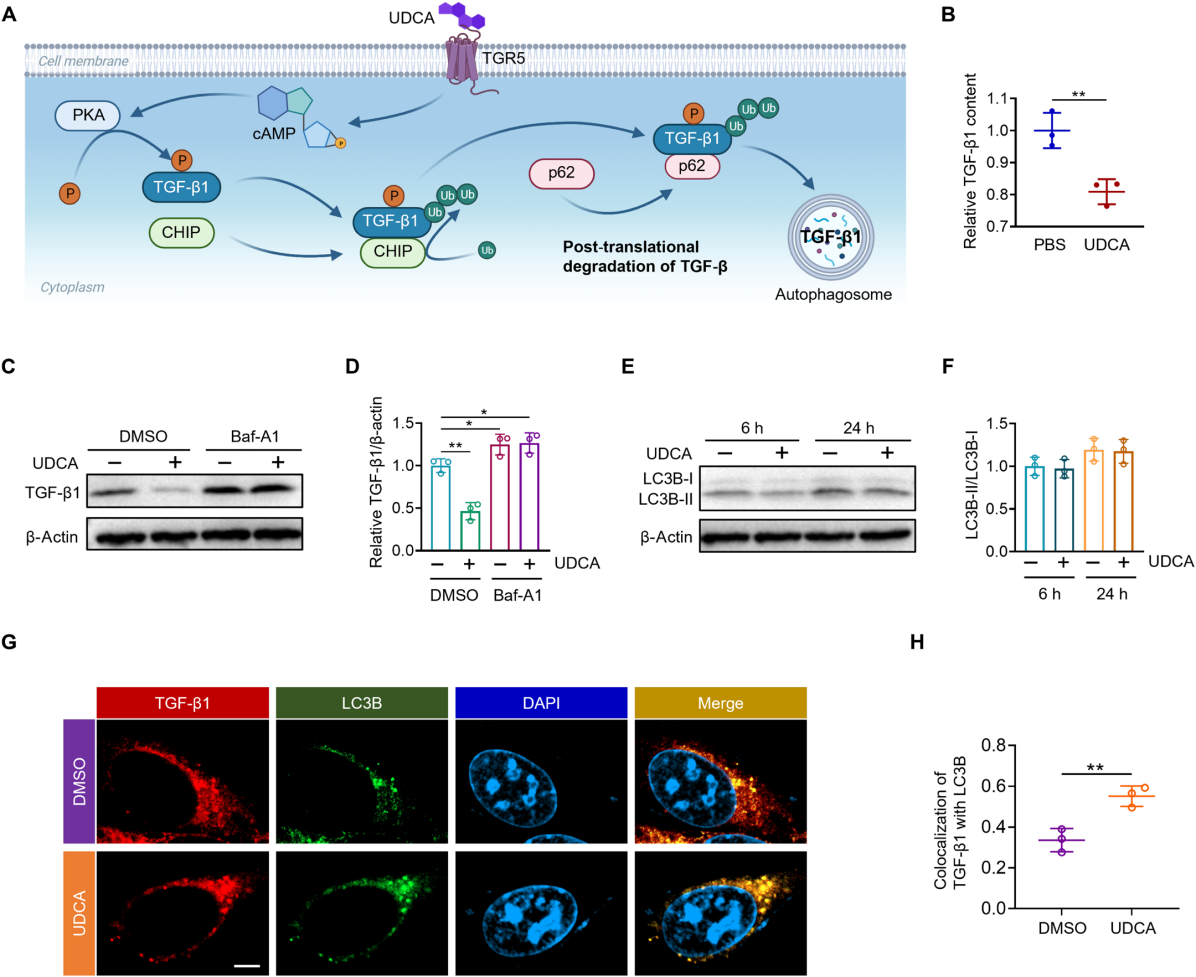
UDCA-mediated TGF-β1 degradation. (**A**) Schematic illustration of the mechanism of UDCA-mediated TGF-β1 degradation. (**B**) ELISA of TGF-β1 in the culture supernatant of B16F10 cells treated with UDCA for 24 hours. (**C**) Western blot analysis of TGF-β1 and β-actin levels of B16F10 cells treated with or without UDCA and Baf-A1 for 24 hours. (**D**) Quantification of immunoblot bands from (C). (**E**) Western blot analysis of LC3B-I and LC3B-II levels of B16F10 cells treated with or without UDCA for the indicated times. (**F**) Quantification of the ratio of LC3B-II to LC3B-I bands from (E). h, hours. (**G**) Immunofluorescence analysis of LC3B and TGF-β1 in B16F10 cells exposed with or without UDCA for 24 hours. Scale bar, 5 μm. (**H**) Quantification of colocalization of TGF-β1 with LC3B from (G). Data represent means ± SD. Statistical differences were calculated using Student’s *t* test (*P < 0.05, **P < 0.01).

IL-2 promotes the proliferation of T cells, including both effector T cells that attack tumors and T_reg_ cells that suppress antitumor immune responses. The balance between these cells is crucial in determining the therapeutic outcome of cancer immunotherapy. TGF-β1 crucially regulates T_reg_ cells by promoting their differentiation, stability, and suppressive function (*25*). In advanced cancer stages, TGF-β1 secreted by tumors fosters T_reg_ cell expansion, promoting tumor progression and immune evasion by suppressing the functions of effector T cells (*26*). Recent findings indicate that UDCA induces the degradation of TGF-β1 in CD4^+^ T cells, ultimately suppressing the differentiation of T_reg_ cells (*19*). To verify the degradation capability of UDCA on tumor-related TGF-β1, B16F10 cells were cocultured with 50 μM UDCA for 24 hours, followed by the quantification of TGF-β1. Enzyme-linked immunosorbent assay (ELISA) analysis of the culture supernatant revealed that the administration of UDCA reduced TGF-β1 secretion by 19.1% compared to the control group (Fig. 4B). Western blot analysis revealed that the cells incubated with UDCA decreased the total intracellular TGF-β1 content by 53.5% (Fig. 4, C and D). Previous research has revealed that UDCA regulates TGF-β1 in CD4^+^ T cells through the autophagy pathway (*19*) (Fig. 4A). To investigate whether UDCA also degrades TGF-β1 in tumor cells by regulating autophagy, the autophagy inhibitor bafilomycin A1 (Baf-A1) was used to block autophagy and evaluate its effect on TGF-β1 levels. As demonstrated in Fig. 4 (C and D), Baf-A1 reversed the reduction of TGF-β1 by UDCA, confirming the involvement of autophagy in this process. We further examined the expression of autophagy markers in UDCA-treated B16F10 cells, including LC3B-I and LC3B-II. The results showed that UDCA had no regulatory effect on the ratio of LC3B-II/LC3B-I (Fig. 4, E and F). However, dual immunofluorescence staining of TGF-β1 and LC3B indicated that UDCA increased the colocalization of TGF-β1 in autophagosomes (Fig. 4, G and H). These data demonstrated that UDCA may induce the posttranslational degradation of TGF-β1 in tumor cells by enhancing its colocalization with autophagosomes. Given UDCA’s inhibitory effect on tumor-associated TGF-β1, it is anticipated to counteract T_reg_ cell activation induced by IL-2, thereby enhancing its antitumor efficacy.

### Injectable ULNPs-cRNA^IL-2F^@G for the treatment of melanoma

**Fig. 5.**
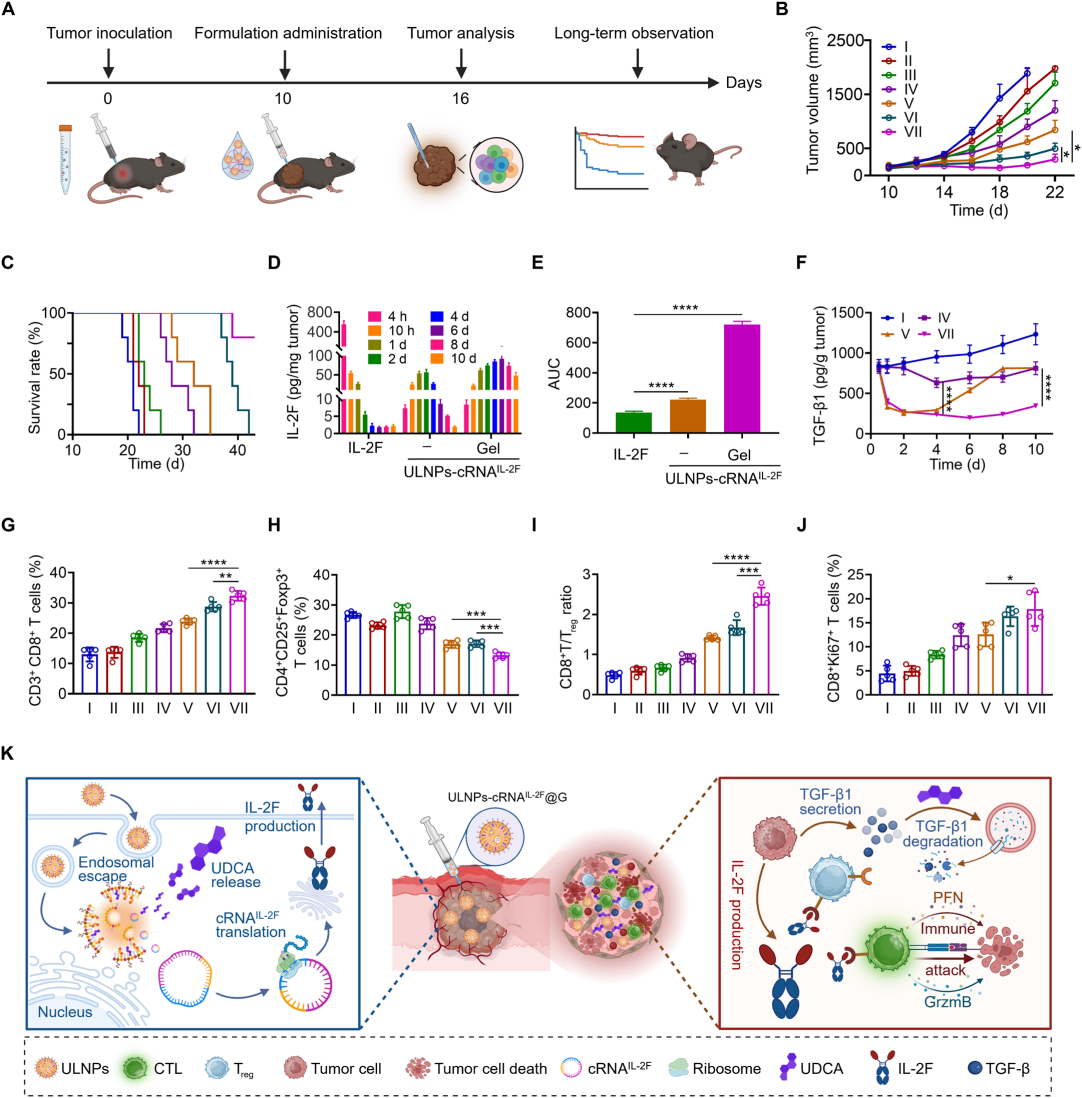
Injectable ULNPs-cRNA^IL-2F^@G suppresses the progression of B16F10 melanoma. (**A**) Schematic illustration of the experimental design. (**B** and **C**) Tumor growth curves (B) and survival curves (C) of the mice after different treatments. (**D**) The level of IL-2F in tumors at different times postinjection. (**E**) AUC of intratumoral IL-2F content from (D). (**F**) The level of TGF-β1 in tumors detected by ELISA. (**G** and **H**) Statistic FCM results of CD8^+^ T cells and T_reg_ cells in tumors among different groups. (**I**) Intratumoral ratio of CD8^+^ T/T_reg_ in tumors from the mice after different treatments. (**J**) The percentage of Ki67^+^ within CD8^+^ T cells using FCM analysis. (**K**) Schematic illustration of antitumor immunotherapy. I, PBS; II, ULNPs@Gel; III, IL-2F; IV, CLNPs-cRNA^IL-2F^; V, ULNPs-cRNA^IL-2F^; VI, CLNPs-cRNA^IL-2F^@G; VII, ULNPs-cRNA^IL-2F^@G. Data represent means ± SD. Statistical differences were calculated using Student’s *t* test (*P < 0.05, **P < 0.01, ***P < 0.001, ****P < 0.0001).

To explore the antitumor efficacy of ULNPs-cRNA^IL-2F^@G, we established a subcutaneous melanoma model in C57BL/6J mice (Fig. 5A). The mice in each group only received a single injection throughout the study duration. As displayed in Fig. 5B and fig. S13, the tumor inhibition rates (TIRs) of mice treated with IL-2F, CLNPs-cRNA^IL-2F^, and ULNPs-cRNA^IL-2F^ were 9.4, 39.8, and 57.9%, respectively, suggesting that the utilization of cRNA^IL-2F^ improved the antitumor efficacy, and the employment of ULNPs as a delivery vehicle further augmented the final therapeutic performances. Excitingly, the mice treated with ULNPs-cRNA^IL-2F^@G exhibited the most potent tumor suppression with a TIR of 85.1%, which was 1.5-fold of that treated with ULNPs-cRNA^IL-2F^, verifying that the incorporation of hydrogel could further promote the antitumor efficacy. Benefiting from the improved antitumor efficacy, the median survival time of mice treated with ULNPs-cRNA^IL-2F^@G was extended to 44 days, which was greatly longer than that of PBS, ULNPs@G, IL-2F, CLNPs-cRNA^IL-2F^, ULNPs-cRNA^IL-2F^, and CLNPs-cRNA^IL-2F^@G (Fig. 5C).

Encouraged by the above results, we further investigated the intratumoral immune responses. Flow cytometry (FCM) results illustrated that the frequency of tumor-infiltrating CD8^+^ T cells was 32.4% for the mice administrated with ULNPs-cRNA^IL-2F^@G, which was higher than that of ULNPs-cRNA^IL-2F^ (23.9%), CLNPs-cRNA^IL-2F^ (21.7%), and IL-2F (18.5%), indicating an enhanced antitumor immunity (Fig. 5G). The administration of ULNPs-cRNA^IL-2F^@G greatly activated the proliferation of CD8^+^ T cells, and the frequency of tumor-infiltrating CD8^+^ Ki67^+^ T cells reached 17.9% in mice treated with ULNPs-cRNA^IL-2F^@G, surpassing the frequencies observed in ULNPs-cRNA^IL-2F^ (12.6%), CLNPs-cRNA^IL-2F^ (12.4%), and IL-2F (8.4%) groups (Fig. 5J). Because IL-2 plays a crucial role in maintaining the function of CD8^+^ T cells, the reason for the above results may be attributed to varying degrees of CD8^+^ T cell activation by IL-2F. The quantitative detection of intratumoral IL-2F confirmed our speculation. As displayed in Fig. 5D, the administration of IL-2F exhibited a rapid peak in intratumoral IL-2F level at 4 hours postinjection, followed by a sharp decline, which led to the ineffective activation of CD8^+^ T cells. In comparison, ULNPs-cRNA^IL-2F^ greatly prolonged the half-life of IL-2F, and the intratumoral IL-2F level was 1.6 times that of the IL-2F group. Notably, the encapsulation by hydrogel further improved the stability of ULNPs-cRNA^IL-2F^, with its intratumoral IL-2F levels being 5.3 times that of the IL-2F group, which contributed to the most effective and persistent activation of CD8^+^ T cells (Fig. 5E).

As mentioned previously, UDCA had the function of reducing TGF-β1. Figure 5F indicates that the levels of intratumoral TGF-β1 for the mice injected with ULNPs-cRNA^IL-2F^@G were 1.4-fold lower than those of the ULNPs-cRNA^IL-2F^ and CLNPs-cRNA^IL-2F^ on day 10 postinjection. It has been proven that TGF-β1 plays a key role in regulating the generation and function of T_reg_ cells (*27*). Consistent with the trend of TGF-β1, the mice injected with ULNPs-cRNA^IL-2F^@G exhibited the lowest T_reg_ cell proportion (13.2%) compared to those injected with ULNPs-cRNA^IL-2F^ (16.9%) and CLNPs-cRNA^IL-2F^ (23.8%) (Fig. 5H). These data showed that hydrogel incorporation enabled sustained local release of ULNP, prolonging their effects at the tumor site and greatly inhibiting T_reg_ cells. The elevated ratio of CD8^+^ T/T_reg_ is widely acknowledged as an indicative marker of favorable immune response against tumors. As shown in Fig. 5I, the mice injected with ULNPs-cRNA^IL-2F^@G demonstrated the highest CD8^+^ T/T_reg_ ratios (2.5) compared to those injected with IL-2F (0.7), CLNPs-cRNA^IL-2F^ (0.9), ULNPs-cRNA^IL-2F^ (1.4), and CLNPs-cRNA^IL-2F^@G (1.7). In line with the FCM results, immunofluorescence images revealed that the administration of ULNPs-cRNA^IL-2F^@G induced a large expansion of perforin and granzyme B, which were cytotoxic molecules secreted by cytotoxic T lymphocytes, indicating the robust antitumor T cell immune responses (fig. S23).

### Lyophilized ULNPs-cRNA^IL-2F^@G for the treatment of postsurgical melanoma

**Fig. 6.**
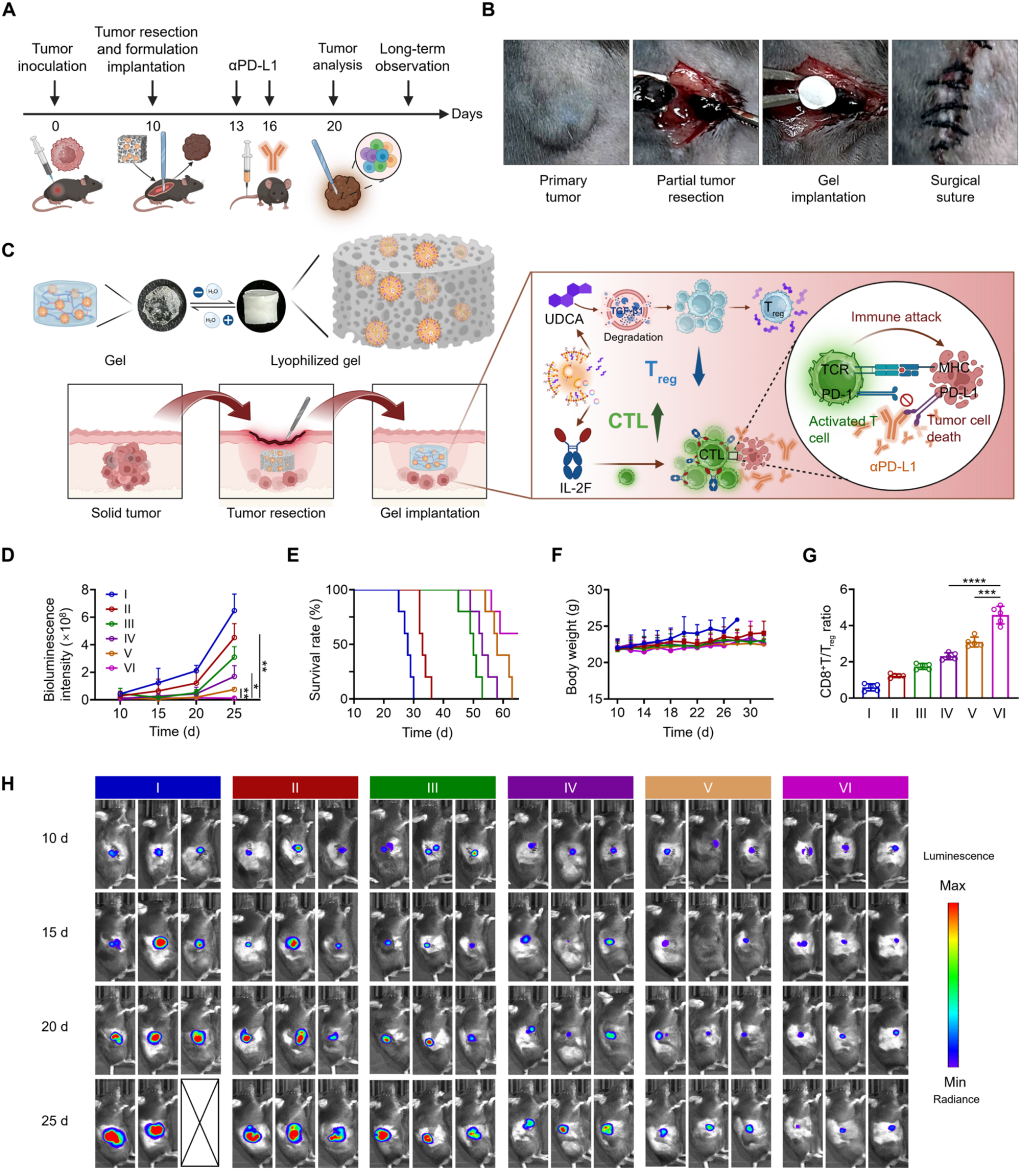
Lyophilized ULNPs-cRNA^IL-2F^@G for postsurgical melanoma treatment. (**A**) The time course of the study. (**B**) Intraoperative view of partial tumor resection and lyophilized hydrogel implantation. (**C**) The mechanism of ULNPs-cRNA^IL-2F^@LG and αPD-L1 for the synergetic immunotherapy of postresection melanoma. (**D**) The quantitative analysis of bioluminescence imaging in (H). (**E**) Survival curves of the mice after different treatments. (**F**) Body weight of mice after different treatments. (**G**) FCM analysis of the intratumoral ratio of CD8^+^ T/T_reg_. (**H**) In vivo bioluminescence imaging of the mice after different administrations. I, PBS; II, αPD-L1; III, CLNPs-cRNA^IL-2F^@LG; IV, ULNPs-cRNA^IL-2F^@LG; V, CLNPs-cRNA^IL-2F^@LG + αPD-L1; VI, ULNPs-cRNA^IL-2F^@LG + αPD-L1. Data represent means ± SD. Statistical differences were calculated using Student’s *t* test (*P < 0.05, **P < 0.01, ***P < 0.001, ****P < 0.0001).

Surgical resection remains the preferred treatment for solid tumors, but postoperative tumor recurrence is common in clinical practice and greatly impairs the prognosis of cancer. Considering that lyophilized hydrogel (LG) is more convenient for surgical operations, it is highly promising for intraoperative implantation to prevent postoperative recurrence. To further assess the potency of ULNPs-cRNA^IL-2F^@G, we prepared it in lyophilized form and validated its efficacy using a clinically relevant model of postsurgical melanoma recurrence. In brief, 10 days after C57BL/6J mice were inoculated with 1 × 10^6^ luciferase-tagged B16F10 (B16F10-Luc) melanoma cells, we partially removed the tumor and retained approximately 70 mm^3^ of tumor. Meanwhile, the LG was implanted into the surgical resection cavity (Fig. 6, A and B).

Immune checkpoint blockade therapy has emerged as a pivotal approach for treating cancer, frequently combined with other therapies to achieve optimal efficacy. Programmed death-ligand 1 (PD-L1) is one of the major immune checkpoints; a high level of PD-L1 expression is the main factor contributing to the immunosuppressive nature of the tumor microenvironment (TME). To better adapt for clinical application and further boost the antitumor efficacy of ULNPs-cRNA^IL-2F^@LG, anti-PD-L1 antibody (αPD-L1) was intravenously injected on days 13 and 16 for synergistic treatment. FCM results depicted that the ratio of tumor-infiltrating CD8^+^ T/T_reg_ was 4.6 for the ULNPs-cRNA^IL-2F^@LG + αPD-L1 treatment, which was much higher than those of ULNPs-cRNA^IL-2F^@LG (2.3) and αPD-L1 (1.2) therapies, indicating an enhanced antitumor immunity (Fig. 6G).

The immunofluorescence images of tumors in the ULNPs-cRNA^IL-2F^@LG + αPD-L1 group also revealed increased infiltration of CD8^+^ T cells and decreased infiltration of T_reg_ cells (figs. S24 and S25). Consequently, the mice treated with ULNPs-cRNA^IL-2F^@LG + αPD-L1 exhibited the greatest tumor suppression with a TIR of 98.2%, which was higher than those treated with ULNPs-cRNA^IL-2F^@LG (88.4%) or αPD-L1 (30.2%) alone (Fig. 6D and fig. S26). Correspondingly, the survival time of the mice treated with ULNPs-cRNA^IL-2F^@LG + αPD-L1 was substantially prolonged (Fig. 6E). These results implied that ULNPs-cRNA^IL-2F^@LG could effectively suppress postoperative tumor recurrence, and its combination with αPD-L1 exhibited synergistic antitumor effects.

### Injectable ULNPs-cRNA^IL-2F^@G for postsurgical GBM treatment

Cold tumors are characterized by low immune cell infiltration and an immunosuppressive TME, which makes them less responsive to immunotherapy and presents challenges for effective cancer treatment strategies. ULNPs-cRNA^IL-2F^@G has shown the capacity to enhance the tumor-infiltrating CD8^+^ T cells and effectively mitigate the immunosuppressive TME. These properties make it well-suitable for treating cold tumors such as GBM, which is an aggressive type of brain tumor that is difficult to completely remove through surgery (*28*). Injectable hydrogels, which can be administered via syringes and reshaped to conform to irregular brain wounds, offer potential advantages in the postoperative treatment of GBM.

**Fig. 7.**
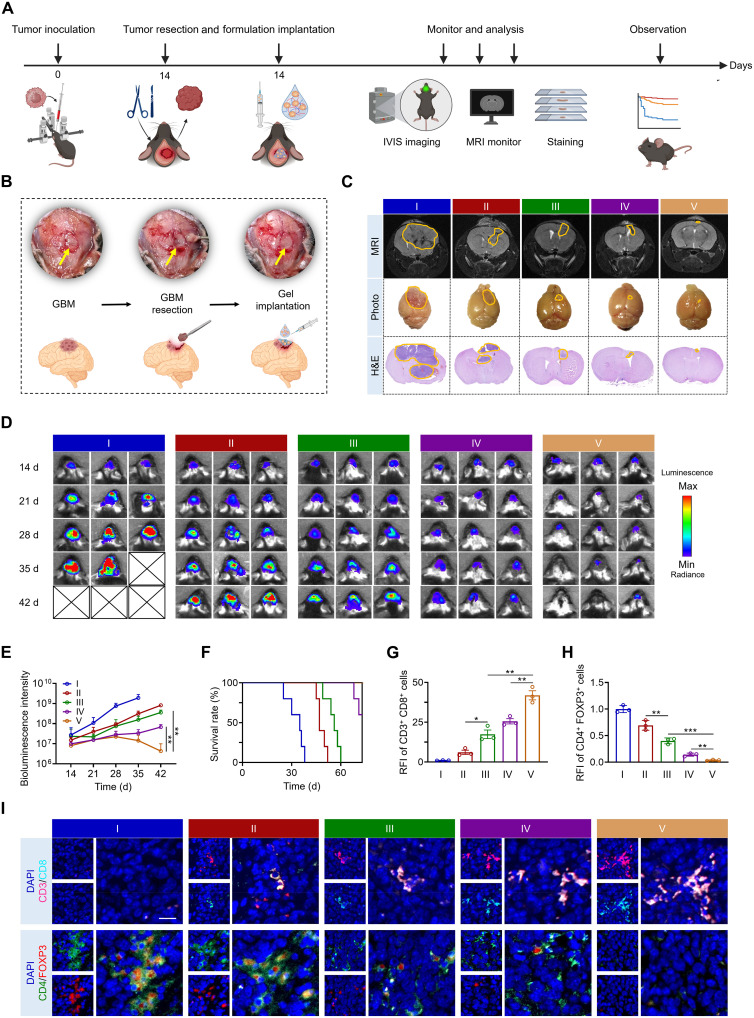
Injectable ULNPs-cRNA^IL-2F^@G for postsurgical glioblastoma treatment. (**A**) Schematic illustration of the treatment schedule. (**B**) Intraoperative view of partial tumor resection and ULNPs-cRNA^IL-2F^@G injection. (**C**) T2W-MRI images, brain photographs, and H&E staining images from each group. Yellow outlines indicate the tumor site. (**D**) In vivo bioluminescence imaging (*n* = 3). (**E**) The quantitative analysis of bioluminescence imaging in (D). (**F**) Survival curves of the mice after different treatments (*n* = 5). (**I**) Immunofluorescence images of tumor-infiltrating CD8^+^ T cells and T_reg_ cells and quantitative analysis of relative fluorescence intensity (**G** and **H**) (*n* = 3). Scale bar, 20 μm. I, PBS; II, CLNPs-cRNA^IL-2F^; III, ULNPs-cRNA^IL-2F^; IV, CLNPs-cRNA^IL-2F^@G; V, ULNPs-cRNA^IL-2F^@G. Data represent means ± SD. Statistical differences were calculated using Student’s *t* test (*P < 0.05, **P < 0.01, ***P < 0.001).

To assess the potency of ULNPs-cRNA^IL-2F^@G in the treatment of postoperative GBM, 2 × 10^5^ luciferase-tagged GL261 cells (GL261-Luc) were intracranially injected into C57BL/6J mice to generate orthotopic GBM. On day 14 following inoculation, the established tumor was partially removed under stereoscope guidance. Meanwhile, ULNPs-cRNA^IL-2F^@G was injected into the resection cavity (Fig. 7, A and B). As shown in Fig. 7 (D and F), the treatment of ULNPs-cRNA^IL-2F^@G led to 99.3% tumor remission, and no mice died during the treatment period. Mice with GBM treated with ULNPs-cRNA^IL-2F^@G showed a notable reduction in bioluminescence intensity, which was 0.7% of PBS-treated mice, 4.6% of CLNPs-cRNA^IL-2F^–treated mice, 9.1% of ULNPs-cRNA^IL-2F^–treated mice, and 43.0% of CLNPs-cRNA^IL-2F^@G–treated mice (Fig. 7E). In line with the results of bioluminescence intensity, T2-weighted magnetic resonance imaging (T2W-MRI), hematoxylin and eosin (H&E) staining images, and brain anatomical photographs also demonstrated that THE ULNPs-cRNA^IL-2F^@G group exhibited the smallest tumor size, suggesting that the treatment of ULNPs-cRNA^IL-2F^@G remarkably inhibited tumor growth and prevented postoperative recurrence of GBM (Fig. 7C).

The proportion of tumor-infiltrating CD8^+^ T cells was highest in mice treated with ULNPs-cRNA^IL-2F^@G, with levels being 0.6-, 1.4-, 5.9-, and 40.9-fold higher than those in mice treated with CLNPs-cRNA^IL-2F^@G, ULNPs-cRNA^IL-2F^, CLNPs-cRNA^IL-2F^, and PBS, respectively (Fig. 7G). In addition, the proportion of tumor-infiltrating T_reg_ cells was lowest in mice treated with ULNPs-cRNA^IL-2F^@G, which was 24.8, 8.8, 5.1, and 3.5% of the levels in mice treated with CLNPs-cRNA^IL-2F^@G, ULNPs-cRNA^IL-2F^, CLNPs-cRNA^IL-2F^, and PBS, respectively (Fig. 7H). These results indicated that ULNPs-cRNA^IL-2F^@G had the ability to reverse the immunosuppressive TME of GBM, converting cold tumors into hot tumors, leading to the effective inhibition of tumor growth. ULNPs-cRNA^IL-2F^@G represented a robust platform for cold tumor immunotherapy, promising to conquer treatment resistance and bolster immune responses in these challenging cases.

Biological safety is the prerequisite for the application of ULNPs-cRNA^IL-2F^@G. We assessed the short-term (24 hours) and long-term (up to 30 days) safety of ULNPs-cRNA^IL-2F^@G by multiple dimensions, such as acute hematological effects, organ impairment, systemic cytokines, and biodegradability. No obvious alterations were observed in blood routine tests between the PBS group and the ULNPs-cRNA^IL-2F^@G group 24 hours after subcutaneous injection, demonstrating that ULNPs-cRNA^IL-2F^@G had no acute toxicity (figs. S27 and S28). As for long-term safety evaluation, mice receiving two doses of subcutaneous injection of the ULNPs-cRNA^IL-2F^@G within 1 month showed comparable levels of blood routine test to those after PBS treatment (fig. S29), suggesting the absence of chronic toxicity. Hepatic/renal function index and systemic cytokine level results indicated that ULNPs-cRNA^IL-2F^@G induced no off-target effects or immune-related adverse events (figs. S31 and 32). Furthermore, no occurrence of skin rash, hair loss, or organ impairment was observed after ULNPs-cRNA^IL-2F^@G treatment (fig. S33). We further investigated the in vivo degradation of the ULNPs-cRNA^IL-2F^@G. As depicted in fig. S34, hydrogel nodules were observed on the 7th day after administration, which were completely degraded 30 days later, indicating the biodegradation capability of the ULNPs-cRNA^IL-2F^@G. Collectively, these results demonstrate the reliable safety of ULNPs-cRNA^IL-2F^@G, guaranteeing its widespread clinical application.

## DISCUSSION

The limitations of conventional IL-2 therapy for tumor treatment include its limited capability to induce durable immune responses due to its short half-life. Although the systematic distribution contributes to immune recruitment, its narrow therapeutic window leads to non-negligible systemic toxicity at high dose (*29*, *30*). Furthermore, IL-2 can activate immunosuppressive T_reg_ cells, which promote immune evasion and progression of tumors (*31*). Current engineering strategies for IL-2 modification include PEGylation, site-specific mutagenesis, fusion proteins, and cytokine combinations; however, these strategies also come with potential drawbacks, such as immunogenicity, drug inactivation, or irreversible protein structural changes. In addition, there may be challenges in achieving an optimal balance between therapeutic efficacy and minimizing side effects. mRNA-based protein replacement therapy holds promise for comprehensive modification. Nevertheless, the available studies on mRNA-based IL-2 therapy all have their limitations. BNT153 (*32*), an mRNA-based IL-2 therapy developed by BioNTech, holds promise for extending the half-life of IL-2 but fails to prevent T_reg_ activation. Another variant, BNT151 (*33*), uses LNPs to encapsulate mRNA encoding mutated IL-2 (enhances its binding to CD122 and fused with albumin), extending the half-life of IL-2 and reducing the nonspecific activation of T_reg_ cells. However, neither of these approaches can prevent IL-2–related systemic toxicity. Further research has explored using polyethyleneimine-modified porous silicon nanoparticles (PPSNs) to encapsulate mRNA encoding mutated IL-2. This approach, combined with intratumoral administration, enhances IL-2 efficacy while reducing systemic toxicity (*34*). However, the low biocompatibility of PPSN hinders its clinical translation.

cRNA has a unique structure without 5′ and 3′ ends, which makes it resistant to degradation by exonucleases (*9*). This increased stability allows cRNA to maintain protein expression for a longer period in the body compared to linear mRNA, thereby enhancing therapeutic efficacy (*10*). In addition, cRNA exhibits lower immunogenicity, making it a safer option for protein replacement therapy (*11*). In this study, we introduce an innovative approach to enhance IL-2 therapy by using immune-regulatory ULNPs to encapsulate cRNA^IL-2F^. These ULNPs-cRNA^IL-2F^ are then incorporated into hydrogels for localized administration in solid tumor therapy. The utilization of cRNA^IL-2F^ enables durable and efficient IL-2F translation, effectively extending its half-life. Moreover, the incorporation of hydrogel facilitates localized and sustained release of ULNPs-cRNA^IL-2F^. When combined with local administration, this approach holds promise for boosting IL-2 efficacy while minimizing systemic toxicity.

Different from the traditional LNPs system, this study develops an immune-regulatory ULNPs. These ULNPs not only effectively encapsulate cRNA^IL-2F^ but also function as TGF-β1 inhibitors, thereby suppressing the activity of T_reg_ cells. The utilization of ULNPs counteracts the nonspecific activation of T_reg_ cells, preventing IL-2–related side effects and enhancing its antitumor efficacy. In contrast to conventional LNPs used merely as carriers for nucleic acid drugs, our innovative integration of UDCA into LNPs endows them with immunomodulatory properties, greatly broadening the scope of LNPs in cancer immunotherapy. Ascribing to the capacity to induce TGF-β1 degradation and suppress T_reg_ function, ULNPs have the potential to be a powerful and versatile carrier of nucleic acid and other therapeutics for immunotherapy against various types of tumors. Particularly in the case of challenging cold tumors with an immunosuppressive TME, characterized by elevated levels of TGF-β1 and T_reg_ cells, ULNPs offer distinctive application potential and may revolutionize the landscape of tumor immunotherapy. In addition, the ULNPs-gel system we develop theoretically has the capability to encapsulate other therapeutic nucleic acids or tumor antigens, as well as to load chemotherapeutic drugs for combination therapy, which warrants further exploration.

The hydrogel exhibits a noteworthy ability to reduce systemic drug concentration while enhancing drug accumulation within the tumor. This dual mechanism effectively inhibits tumor growth without inducing severe side effects, thereby addressing the systemic toxicity associated with IL-2 therapy comprehensively. To stabilize ULNPs, we used BPB and PVA as the matrix and used borate ester structure as cross-linking junctions to form a neutral hydrogel with high biocompatibility. This neutral hydrogel avoids the possible interference of strong positive or negative charges on the LNPs-cRNA complex, thereby ensuring the structural stability of LNPs-cRNA and facilitating sustained expression. In addition, the reversible borate ester bonds within this network allow for degradation within 30 days, enabling the gradual release of LNPs-cRNA and reducing the long-term toxicity of the hydrogel. By adjusting the ratio of BPB to PVA, we modulated the mechanical strength of the hydrogels, ultimately achieving cRNA expression for up to 8 days, with an AUC 2.9 times greater than that of the nonhydrogel group, which lays the groundwork for the prolonged efficacy of IL-2. Moreover, the hydrogel exhibits excellent drug-loading capacity and can serve as a platform for loading other therapeutics to achieve safer and more effective anticancer therapies.

In conclusion, this study proposed an improved IL-2 therapy based on ULNPs-cRNA^IL-2F^@G. This therapeutic modality achieved dual immunomodulation by simultaneously activating CD8^+^ T cells while suppressing T_reg_ cells, greatly enhancing the antitumor immune response and inhibiting tumor growth. Furthermore, the sophisticated design markedly diminished IL-2–related systemic toxicity, rendering it a promising option for cancer immunotherapy. The satisfactory safety and ease of formulation also added to the attraction of this improved IL-2 therapy. Overall, the innovative IL-2 therapy we proposed solved the dilemma faced by conventional IL-2 therapy, expanding the application of IL-2 therapy in the field of tumor immunology and holding clinical translational value.

## MATERIALS AND METHODS

### Mice and cell lines

Six-week-old male C57BL/6J mice were purchased from Beijing Weitong Lihua Experimental Animal Technology Co. Ltd. All animal procedures were performed in accordance with the Guidelines for the Care and Use of Laboratory Animals of Tsinghua University and approved by the Animal Ethics Committee of Tsinghua University. The assigned approval/accreditation number is 21-YGC1. Murine melanoma B16F10 or luciferase-labeled B16F10 cells (B16F10-Luc) were gifted by X. Lin at Tsinghua University. Murine GBM luciferase-labeled GL261 cells (GL261-Luc) were gifted by R. Kuai at Tsinghua University. Human embryonic kidney (HEK) 293T cells were purchased from the American Type Culture Collection. All the cells were maintained in Dulbecco’s modified Eagle’s medium containing 10% FBS and penicillin-streptomycin (100 U/ml) solution. All the cells were cultured in 5% CO_2_ at 37°C.

### Materials and reagents

Polyvinyl alcohol-1799 (PVA-1799), polyethylene glycol, molecular weight 2000 (PEG-2000, *M*_w_ = 2000), 1,1′-carbonyldiimidazole (CDI), triethylamine, (4-aminophenyl)boronic acid hydrochloride, *N*,*N*-dimethylformamide (DMF), UDCA, and cholesterol were purchased from J&K, TCI, and Sigma-Aldrich. 1,2-Distearoyl-sn-glycero-3-phospho-choline (DSPC), PEG-lipid, (ALC-0159), and ALC-0315 were purchased from Xiamen Sinopeg Biotech Co. Ltd. 4′,6-Diamidino-2-phenylindole (DAPI) was purchased from Beyotime. All reagents were commercially available and used as supplied without further purification. Solvents were used as purchased or dried according to procedures described in the literature. Information on all the antibodies used in this study is provided in table S1.

### Animal models

A total of 5 × 10^5^ B16F10 cells were subcutaneously inoculated into the right flank of the C57BL/6J mice to establish a melanoma mouse model. Ten days after inoculation, mice were randomly divided into seven groups and experienced intratumoral injection with PBS, ULNPs@Gel, IL-2F (2 μg per mouse), CLNPs-cRNA^IL-2F^ (2 μg cRNA^IL-2F^ per mouse), ULNPs-cRNA^IL-2F^ (2 μg cRNA^IL-2F^ per mouse), CLNPs-cRNA^IL-2F^@G (2 μg cRNA^IL-2F^ per mouse), and ULNPs-cRNA^IL-2F^@G (2 μg cRNA^IL-2F^ per mouse). All groups were administered once throughout the study period.

The postoperative melanoma mouse model was established as follows: Mice bearing the subcutaneous B16F10 melanoma model were treated as described above. Ten days after inoculation, we partially removed the tumor, leaving approximately 70 mm^3^ of tumor tissue, and simultaneously implanted the LG. The combination therapy group received 20 μg of αPD-L1 per mouse through the tail vein on days 13 and 16. The tumor growth was examined by IVIS (IVIS Lumina III, Caliper Life Sciences) and data were analyzed by Living Image software (PerkinElmer).

The postoperative GBM mouse model was established as follows: 2 × 10^5^ GL261-Luc cells in 7 μl of PBS were intracranially injected into C57BL/6J mice (1.8 mm lateral and 1 mm posterior to the bregma, 2 mm of depth) to generate the orthotopic GBM model. On day 14 following inoculation, mice were anesthetized with Avertin (300 mg/kg, intraperitoneal injection) and fixed in a stereotaxic apparatus. The skull of the mice was surgically exposed, and a hole was drilled to allow for tumor resection. After 80% of the primary tumor was resected under stereoscope guidance, the mice were divided into five groups and injected with PBS, CLNPs-cRNA^IL-2F^, ULNPs-cRNA^IL-2F^, CLNPs-cRNA^IL-2F^@G, or ULNPs-cRNA^IL-2F^@G (2 μg cRNA^IL-2F^ per mouse). The tumor growth of these mice was examined weekly through IVIS. On day 35, in vivo MR imaging, H&E staining, and immunofluorescence staining were conducted to analyze the tumor.

### Preparation and characterization of ULNPs

ALC-0315, cholesterol, ALC-0159, and DSPC were dissolved in ethanol with molar ratios of 48.0, 40.0, 2.0, and 10.0, respectively. cRNA^IL-2F^ was dissolved in a 100 mM citrate buffer at pH 5.0. The lipid mixture and the solution of cRNA^IL-2F^ were mixed at a volume ratio of 1:3 with a weight ratio of 20:1 (total lipids/cRNA), followed by dialysis against PBS for 2 hours for further use. ULNPs were prepared using a molar ratio of 12.0%/48.0%/28.0%/2.0%/10.0% for UDCA, ALC-0315, cholesterol, ALC-0159, and DSPC, respectively. The hydrodynamic size and zeta potential of ULNPs were measured by Zetasizer Nano ZS90 (Malvern Instruments, Malvern). Cryo-TEM was used to image the ULNPs. Three microliters of the sample solution was dropped on a carbon-coated copper grating and dried at room temperature. FEI Talos 200 C Field Emission Gun high-resolution TEM was used in this study.

To verify the translation efficiency of ULNPs, B16F10 cells were incubated with CLNPs-cRNA^EGFP^/ULNPs-cRNA^EGFP^ for 24 hours. Then, the nucleus was stained with Hoechst 33342. CLSM images were recorded on an Olympus FV3000 microscope (Olympus, Nagano, Japan). Rhodamine 6G was loaded into the ULNPs to monitor the lysosomal escape of ULNPs-cRNA^IL-2F^. Specifically, B16F10 cells were incubated with Rhodamine 6G–labeled ULNPs-cRNA^IL-2F^ for 8 hours. Before CLSM imaging, lysosomes and nuclei were stained with Lyso-Tracker Green and Hoechst 33342 according to the manufacturer’s directions. CLSM images were recorded using a Zeiss LSM980 laser scanning confocal microscope (Zeiss, Oberkochen, Germany).

### Synthesis of BPB

The synthetic route is shown in fig. S1. Briefly, PEG-2000 (2.00 g, 2.00 mM) and CDI (486 mg, 3.00 mM) were dissolved in 30 ml of dichloromethane (DCM) and stirred at room temperature for 24 hours. After the reaction was complete, the solution was concentrated under a vacuum and added dropwise into Et_2_O (50 ml), and the resulting precipitate was centrifuged (2 min, 3000 rpm). The precipitate was redissolved by DCM and precipitated by Et_2_O three times. The final product, PEG-1, was dried in a vacuum oven overnight, yielding a white solid powder (1.82 g, yield: 83.2%). The ^1^H NMR spectrum is shown in fig. S2. PEG-1 (1.09 g, 500 μM), (4-aminophenyl)boronic acid hydrochloride (260 mg, 1.50 mM), and triethylamine (277 μl, 2.00 mM) were dissolved in 10 ml of DMF and stirred at 50°C for 24 hours. After the reaction was complete, the solution was added dropwise into Et_2_O (50 ml), and the resulting precipitate was centrifuged (2 min, 3000 rpm). The precipitate was redissolved by DCM and precipitated by Et_2_O three times. The final product, BPB, was dried in a vacuum oven overnight, yielding a white solid powder (854 mg, yield: 73.4%). The ^1^H NMR spectrum is shown in fig. S3.

### Preparation and characterization of hydrogel

BPB was dissolved in distilled water to form a 5.0% (w/w) solution. PVA-1799 was dissolved in distilled water to form a 2.5% (w/w) solution. Different volumes of PVA solution, ULNPs-cRNA^Luc^ solution, and distilled water were mixed, after which the BPB solution was added to prepare the ULNPs-cRNA^Luc^@G. The volumes of PVA solution, ULNPs-cRNA^Luc^ solution, distilled water, and BPB solution are shown in fig. S7. For the preparation of LG, the hydrogel was rapidly frozen in liquid nitrogen and lyophilized with a vacuum lyophilizer. The morphological properties of LG were conducted using a scanning electron microscope (SU8000, HITACHI, Japan). The rheological properties of hydrogel were recorded by an oscillatory rheometer Kinexus Pro^+^ rheometer (Malvern Instruments).

### cRNA^IL-2F^ synthesis

RNA structure was predicted using RNAFold. cRNA^IL-2F^ was synthesized according to previous reports (*35*). In brief, cRNA^IL-2F^ precursors were synthesized via in vitro transcription (IVT) from the linearized cRNA^IL-2F^ plasmid templates with the T7 High Yield RNA Synthesis Kit (New England Biolabs). cRNA^IL-2F^ was purified by digestion with DNase I at 37°C for 15 min followed by precipitation with lithium chloride. To catalyze the cyclization of cRNA^IL-2F^, GTP (2 mM) was added to the reaction, and the solution were incubated at 55°C for 15 min in T4 RNA Ligase reaction buffer (NEB). Products were then purified with the Monarch RNA Cleanup Kit (New England Biolabs) according to the manufacturer’s protocols. The column-purified RNA was heated at 65°C for 3 min and then immediately placed on ice, after which the RNA samples were digested by RNase R (Epicenter) at 37°C for 15 min to further enrich the cRNA^IL-2F^. The RNA treated with RNase R was subjected to column purification, followed by agarose gel electrophoresis. To optimize the IVT reaction, cRNA^IL-2F^ was directly column purified after IVT and further purified on an Agilent 1100 Series HPLC (Agilent). RNA samples were reverse transcribed to cDNA and amplified by polymerase chain reaction (PCR) with junction-spanning primers, followed by Sanger sequencing. All the coding sequences are supplied in table S2.

### FCM analysis

Table S1 lists the antibodies used for FCM analysis. Tumor cell suspensions were prepared by grinding tumor tissues in PBS containing 1% FBS. Tumor single-cell suspension was prepared by passing homogenates through a 70-μm mesh nylon cell strainer. All of the antibody staining followed the manufacturer’s instructions. Stained cells were measured on a CytoFLEX flow cytometer (Beckman Coulter) and data were analyzed by CytoFLEX Software (Beckman Coulter).

### In vivo MR imaging

T2W-MRI of the brain was performed using an MRI scanner (Burke, 9.4 T). The measurement parameters of T2W-MRI were as follows: repetition time (TR)/echo time (TE) = 3050/40 ms, number of slices = 22, slice thickness = 0.3 mm, echo spacing = 3.569 ms, field of view = 30 × 30 mm, matrix = 240 × 256, repetition time = 2000 ms, average = 3.

### Statistics

Data are presented as means ± SD. The Student’s two-tailed unpaired *t* test was carried out for the two-group comparison, whereas differences among multiple groups were evaluated using one-way analysis of variance (ANOVA). The level of significance was defined at *P < 0.05, **P < 0.01, ***P < 0.001, and ****P < 0.0001.
