# Supplementary material for: Coordinating interleukin-2 encoding circRNA with immunomodulatory lipid nanoparticles to potentiate cancer immunotherapy
Source: Sci Adv. 2025 Feb 26;11(9):eadn7256. doi: 10.1126/sciadv.adn7256 (PMC11864171; doi:10.1126/sciadv.adn7256)
Supplement: Supplementary file 1 — Supplementary Methods Figs. S1 to S36 Tables S1 and S2 [file sciadv.adn7256_sm.pdf]

Science Advances
AAAS

Supplementary Materials for

Coordinating interleukin-2 encoding circRNA with immunomodulatory lipid nanoparticles to potentiate cancer immunotherapy

Kai Yang *et al.*

Corresponding author: Zhida Liu, zhida_liu@saari.org.cn; Guocan Yu, guocanyu@mail.tsinghua.edu.cn

Sci. Adv. **11**, eadn7256 (2025)
DOI: 10.1126/sciadv.adn7256

This PDF file includes:

Supplementary Methods
Figs. S1 to S36
Tables S1 and S2

Methods

Enzyme-linked immunosorbent assay (ELISA). B16F10 cells were exposed to 50 μM of UDCA. After 24 h, TGF-β1 was measured in the culture supernatant by ELISA. Serum and tumor tissues were collected from each group. The following measurements of secreted cytokines by ELISA were conducted according to the manufacturer's instructions (IL-2, TGF-β1, TNF-α and IFN-γ ELISA Kit, Elabscience).

Hematoxylin and eosin staining. Hematoxylin and eosin (H&E) staining of main organs (heart, kidneys, liver, spleen, lungs) was performed after subcutaneous injection of PBS/ULNPs-cRNA^{IL-2F}@G for 2 weeks. H&E staining of brain tissues were performed at 35 d after different treatments.

Western blot assay. Western blot analysis was performed on TGF-β1 after treating B16F10 cells with 50 μM UDCA or 10 nM Baf-A1 for 24 h. Western blot analysis of LC3B-I and LC3B-II was conducted after treating B16F10 cells with or without 50 μM UDCA for the specified durations. Proteins were extracted with RIPA buffer containing protease and phosphatase inhibitors. The concentration of protein was quantified using a bicinchoninic acid assay protein assay kit. Equivalent amounts of total protein were separated by SDS-PAGE and transferred onto PVDF membranes. Block the PVDF membranes with a blocking buffer (5% non-fat milk in TBST) for 2 h at room temperature. Subsequently, the membrane was incubated with a primary antibody specific to the target protein overnight at 4°C. After washed with TBST 3 times, the corresponding secondary antibodies were incubated for 2 h at room temperature. Then, the membranes were washed with TBST 3 times and subjected to enhanced chemiluminescence. Finally, the membrane was imaged using a chemiluminescent imaging system. Western blot bands were quantified with Image J.

Immunofluorescence staining. B16-F10 cells were pretreated with or without 50 μM of UDCA for 24 h before immunofluorescence staining. Fix the cells with paraformaldehyde (4%) for 5 min at room temperature. After being washed with PBS 3 times, the cells were permeabilized using a permeabilization buffer (0.1% Triton X-100 in PBS) for 5 min. Before staining, 5% bovine serum albumin (BSA) was used to block the nonspecific binding. Subsequently, the cells were further incubated with the primary antibody overnight at 4°C. After washed with PBS 3 times, the cells were incubated with corresponding secondary antibodies for 2 h at room temperature. The nuclei were stained with DAPI according to the manufacturer's instructions.

For tumor staining, tumors were extracted and fixed in paraformaldehyde (4%) for 24 h and then dehydrated with ethanol and embedded in paraffin. Paraffin blocks were sectioned by a microtome (Leica, Germany) into 5 μm sections. Before staining, the tissue slides were treated by the following sequence: deparaffinization, rehydration, and antigen retrieval. Then the tissue slides were incubated with 5% BSA to block the nonspecific binding. The tumor slices were stained with the corresponding antibody for 1 h at room temperature. The nuclei were stained with DAPI according to the manufacturer's instructions. The stained slides were imaged by confocal microscopy (LSM 980, Carl Zeiss, Germany) and analyzed with Zen 3.3 and Image J software.

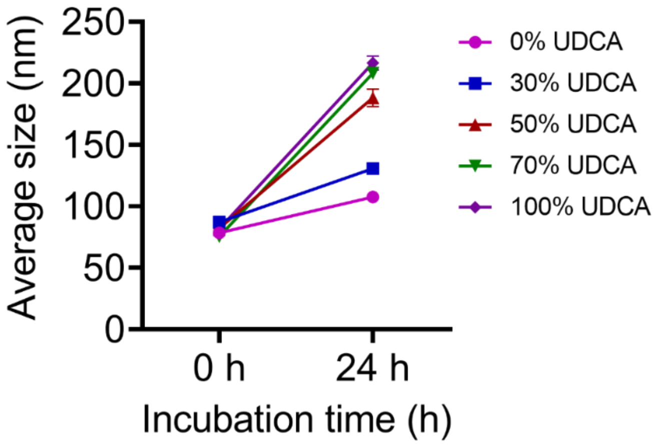

Figure S1. DLS results of ULNPs containing different proportions of UDCA before and after incubation in FBS (10%) for 24 hours. Data are presented as the mean ± SD.

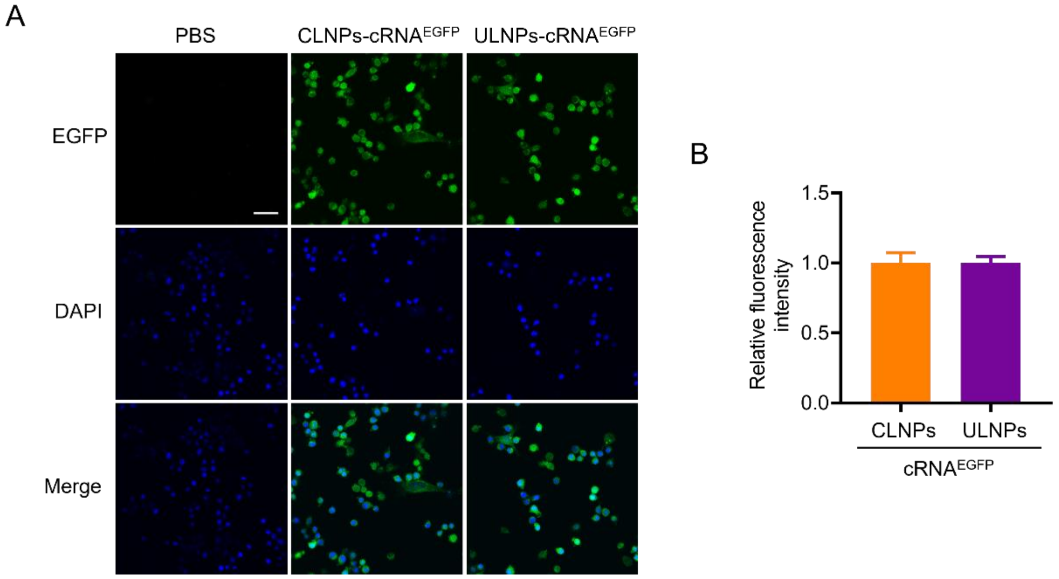

Figure S2. (**A**) CLSM images of B16F10 cells after transfected with CLNPs-cRNA^{EGFP} or ULNPs-cRNA^{EGFP} for 24 h. Scale bar: 50 μm. (**B**) Quantitative analysis of mean fluorescence intensity from (**a**). Data are presented as the mean ± SD.

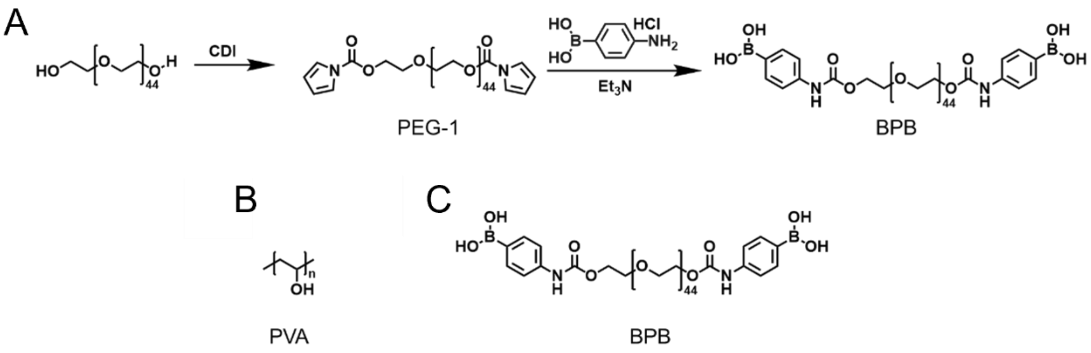

Figure S3. (**A**) Synthetic route of BPB. (**B** and **C**) Chemical structures of PVA (**B**) and BPB (**C**).

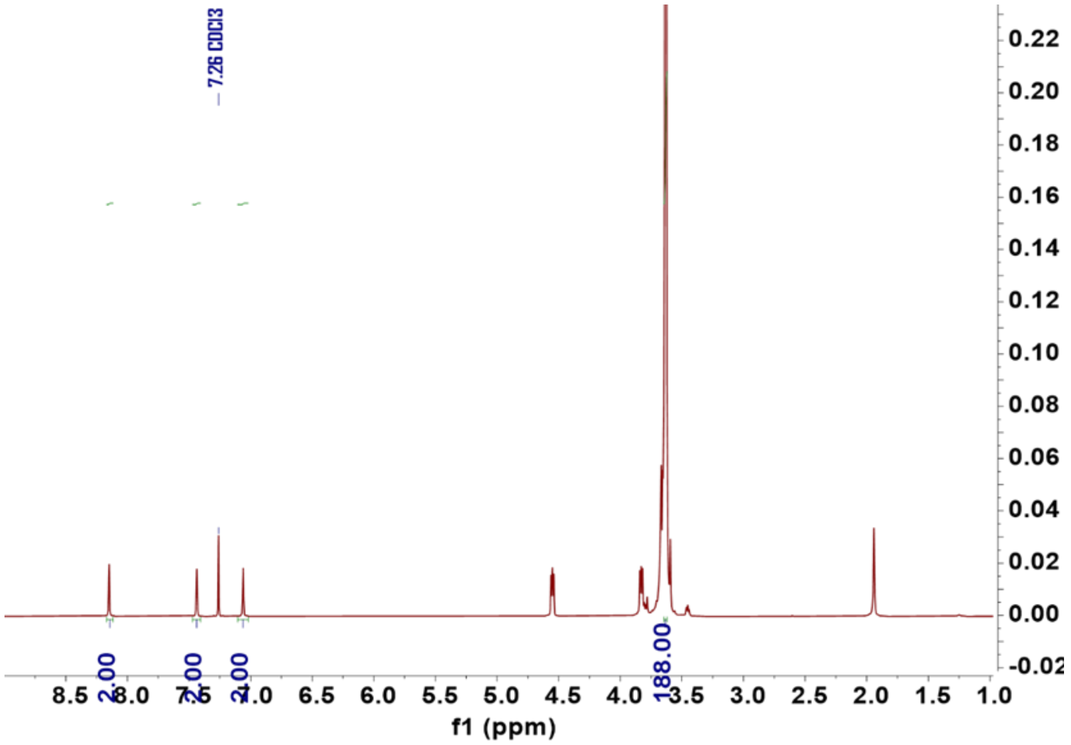

Figure S4. ^{1}H NMR spectrum (400 MHz, $CDCl_3$, room temperature) of compound **PEG-1**.

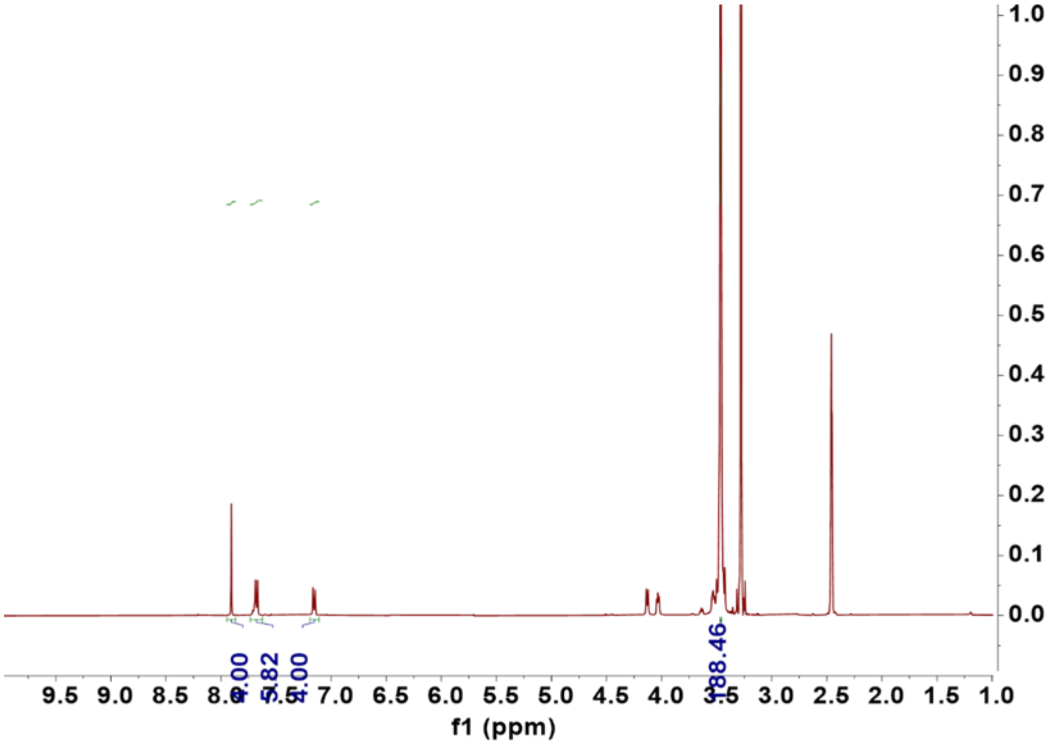

Figure S5. ^{1}H NMR spectrum (400 MHz, DMSO-d_6, room temperature) of compound **BPB**.

	Cholesterol	UDCA
Structure	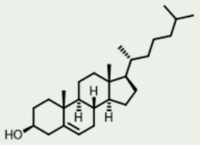	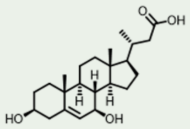
Molecular formula	$C_{27}H_{46}O$	$C_{24}H_{40}O_4$
Molecular weight	386.66	392.57
Water solubility	0.095 mg/L	20 mg/L
Biological function	The main component of biomembrane	Gallstone solubilizing agent; antitumor immunomodulator

Figure S6. The physicochemical properties of cholesterol and UDCA.

Group	2.5% BPB (μL)	5% PVA (μL)	ULNPs-cRNA^{Luc} (μL)	Total volume (μL)	Administration
**ULNPs-cRNA^{Luc}**	-	-	30	30	s.c.
**ULNPs-cRNA^{Luc} @G1**	50	50	30	130	s.c.
**ULNPs-cRNA^{Luc} @G2**	60	40	30	130	s.c.
**ULNPs-cRNA^{Luc} @G3**	50	60	30	140	s.c.
**ULNPs-cRNA^{Luc} @G4**	60	50	30	140	s.c.
**ULNPs-cRNA^{Luc} @G5**	50	70	30	150	s.c.
**ULNPs-cRNA^{Luc} @G6**	70	50	30	150	s.c.

Figure S7. The component ratios of ULNPs-cRNA^{Luc} and ULNPs-cRNA^{Luc}@G.

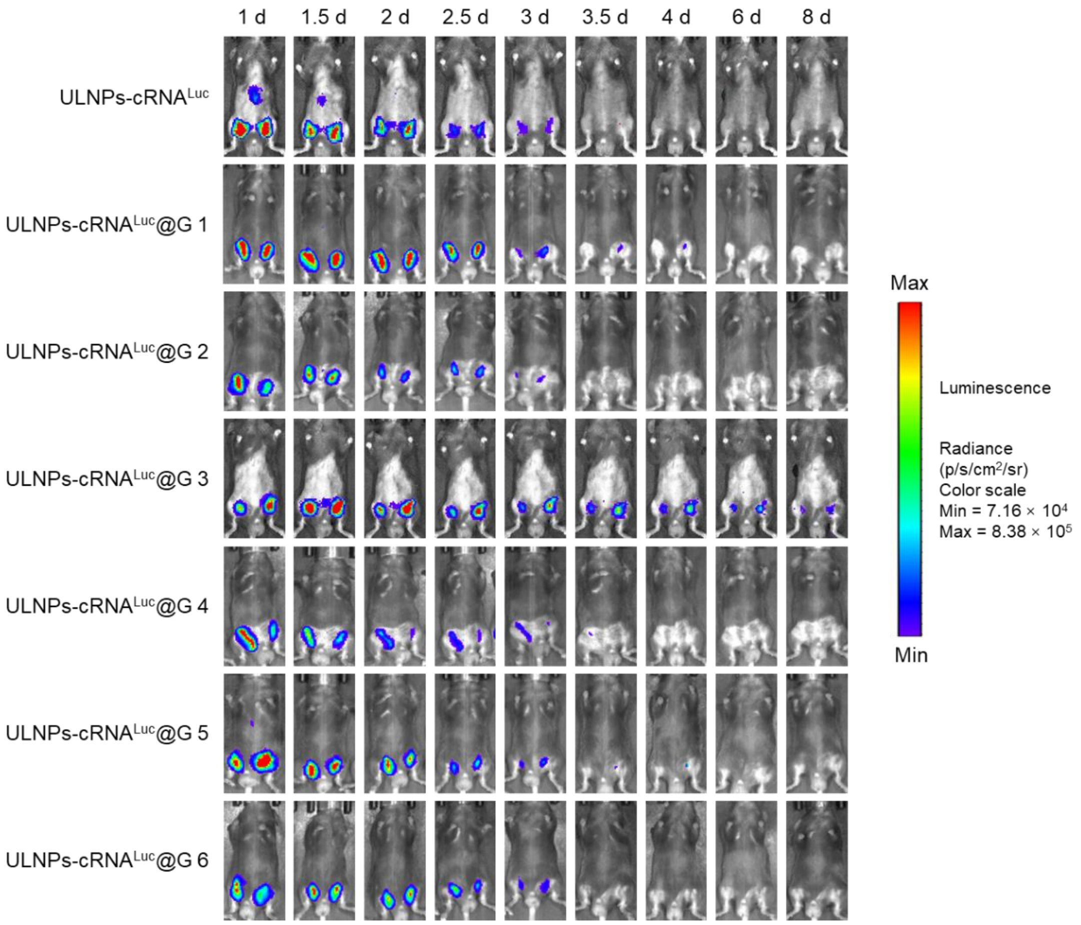

Figure S8. Bioluminescence imaging of the mice subcutaneously injected with ULNPs-cRNA^{Luc} or ULNPs-cRNA^{Luc}@G.

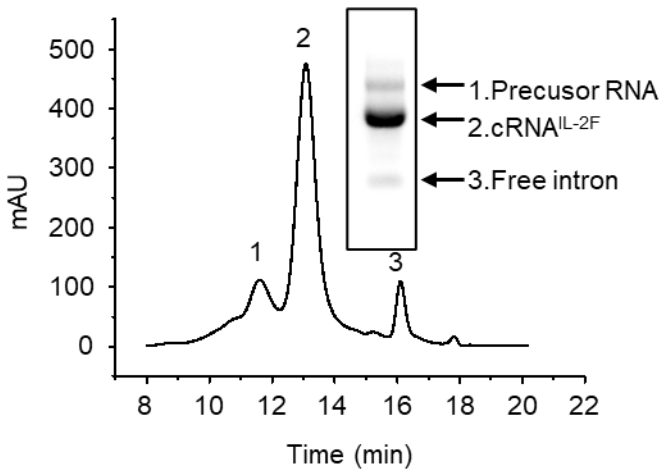

Figure S9. HPLC and agarose gel electrophoresis analysis of cRNA^{IL-2F} from splicing reactions.

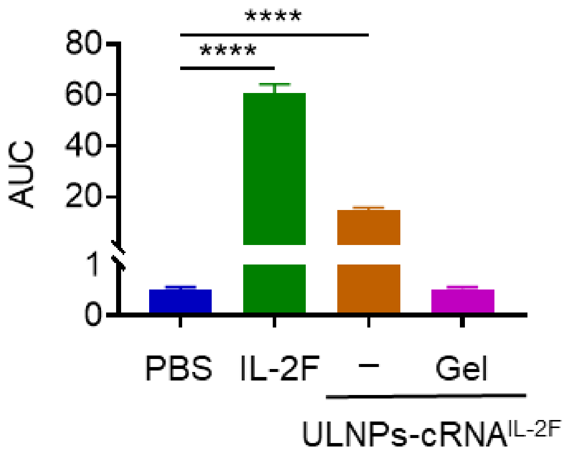

Figure S10. AUC of serum IL-2F content from Figure 2i.

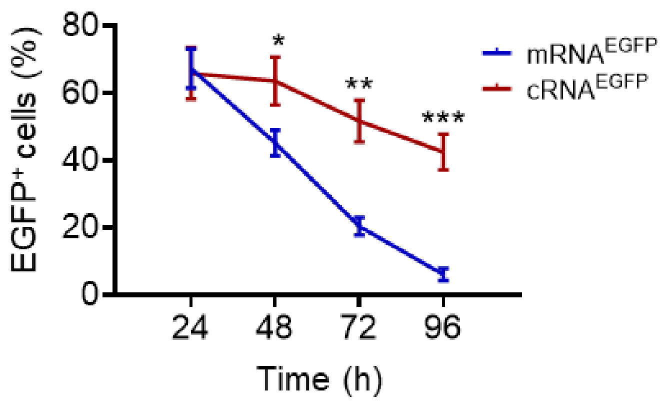

Figure S11. FCM statistical analysis of EGFP-positive HEK293T cells at different time points after transfection.

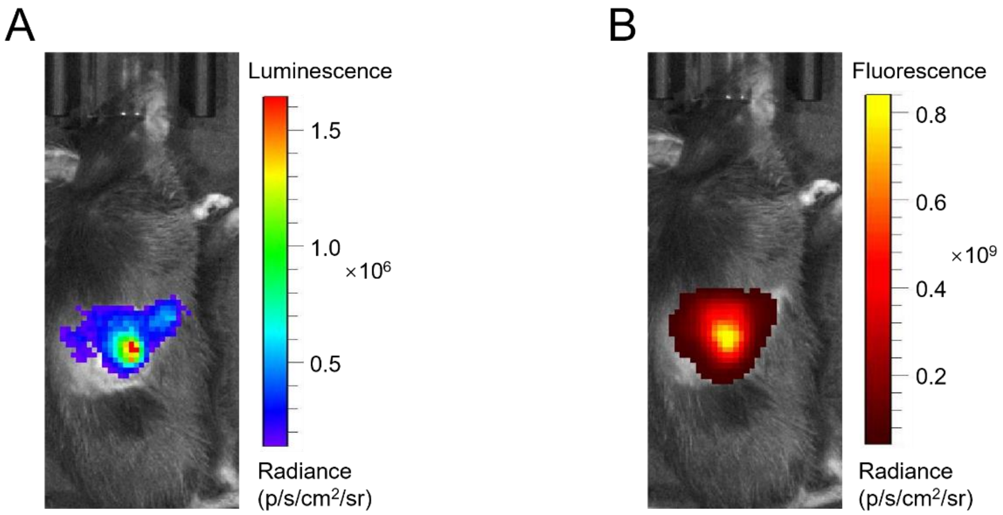

Figure S12. (**A** and **B**) Bioluminescence (**A**) and fluorescence (**B**) imaging of mice at 20 h post intratumoral injection of ULNPs-cRNA^{Luc}@G. DIR was used to label ULNPs.

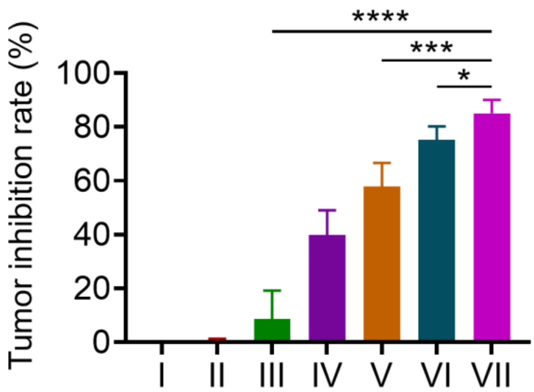

Figure S13. Tumor inhibition rate of the mice after various treatments. I, PBS; II, ULNPs@G; III, IL-2F; IV, CLNPs-cRNA^{IL-2F}; V, ULNPs-cRNA^{IL-2F}; VI, CLNPs-cRNA^{IL-2F}@G; VII, ULNPs-cRNA^{IL-2F}@G. Data represent means ± SD. Statistical differences were calculated using a student's *t* test (*$p < 0.05$, **$p < 0.01$, ***$p < 0.001$, ****$p < 0.0001$).

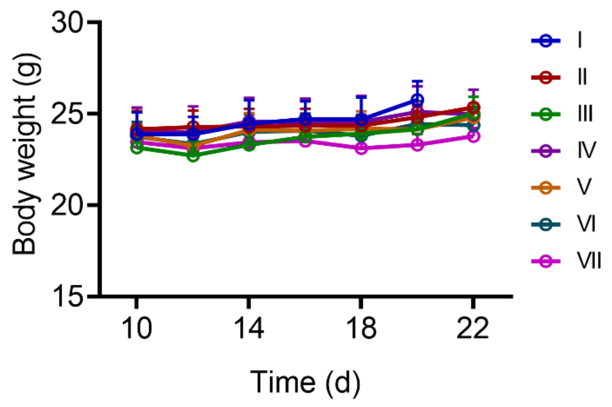

Figure S14. Body weight of mice after different treatments. I, PBS; II, ULNPs@G; III, IL-2F; IV, CLNPs-cRNA^{IL-2F}; V, ULNPs-cRNA^{IL-2F}; VI, CLNPs-cRNA^{IL-2F}@G; VII, ULNPs-cRNA^{IL-2F}@G. Data represent means ± SD.

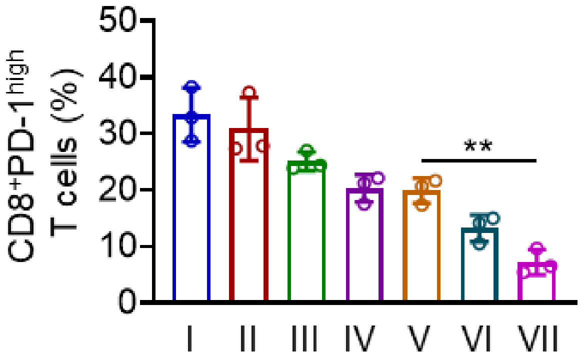

Figure S15. Quantitation of PD-1^{high} within CD8^{+} T cells using FCM analysis. I, PBS; II, ULNPs@G; III, IL-2F; IV, CLNPs-cRNA^{IL-2F}; V, ULNPs-cRNA^{IL-2F}; VI, CLNPs-cRNA^{IL-2F}@G; VII, ULNPs-cRNA^{IL-2F}@G. Data represent means ± SD.

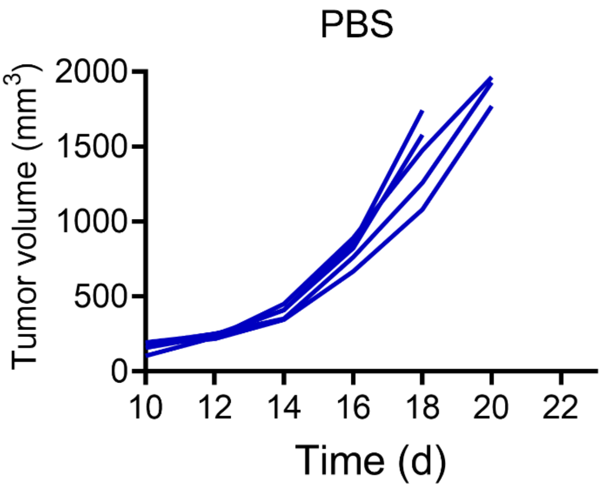

Figure S16. Tumor growth curves of the mice after intratumoral injection of PBS.

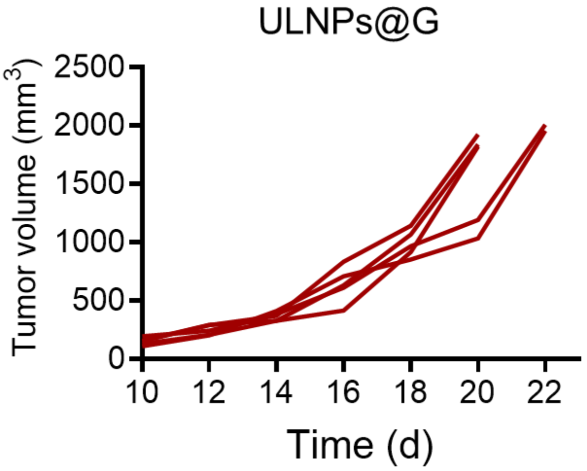

Figure S17. Tumor growth curves of the mice after intratumoral injection of ULNPs@G.

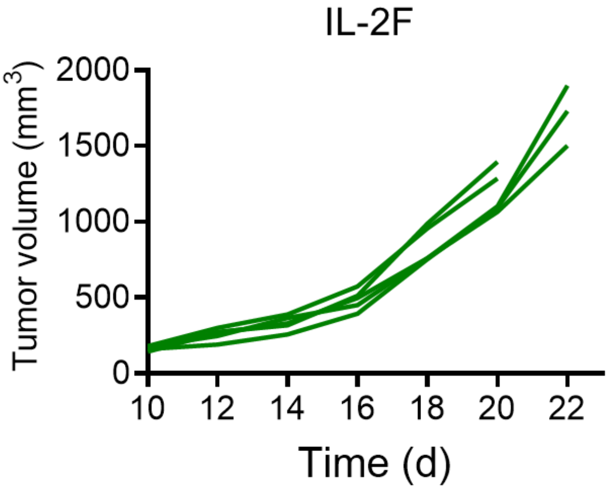

Figure S18. Tumor growth curves of the mice after intratumoral injection of IL-2F.

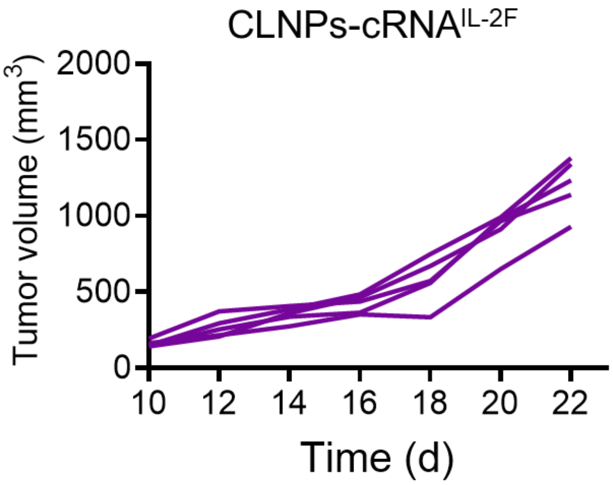

Figure S19. Tumor growth curves of the mice after intratumoral injection of CLNPs-cRNA$^{IL\text{-}2F}$.

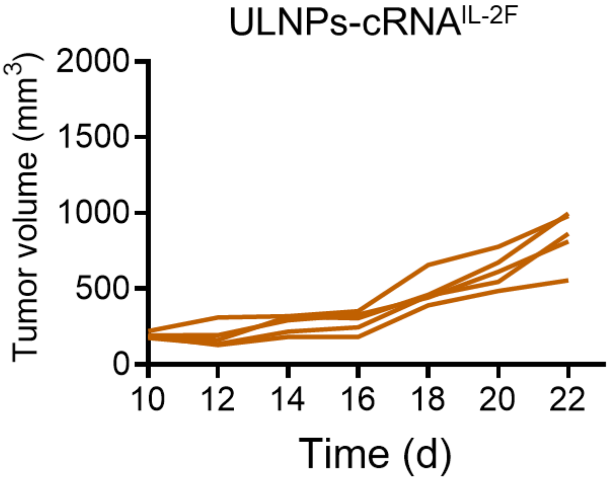

Figure S20. Tumor growth curves of the mice after intratumoral injection of ULNPs-cRNA^{IL-2F}.

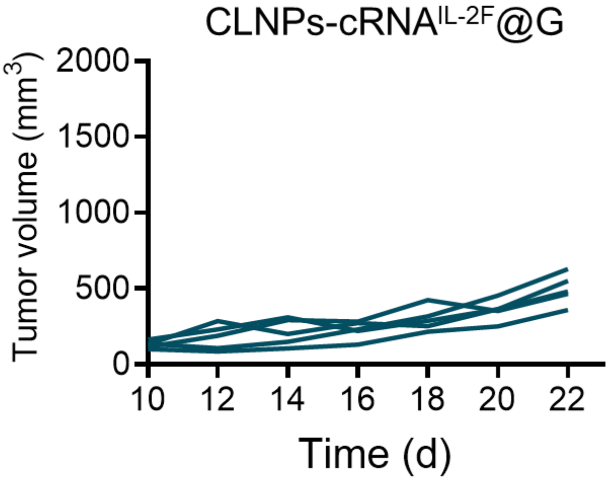

Figure S21. Tumor growth curves of the mice after intratumoral injection of CLNPs-cRNA^{IL-2F}@G.

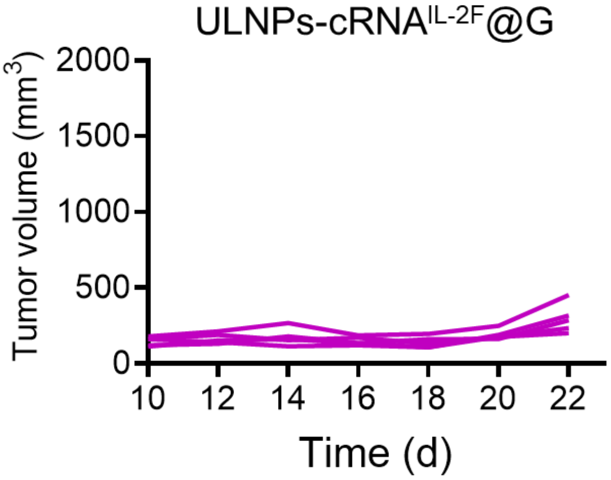

Figure S22. Tumor growth curves of the mice after intratumoral injection of ULNPs-cRNA^{IL-2F}@G.

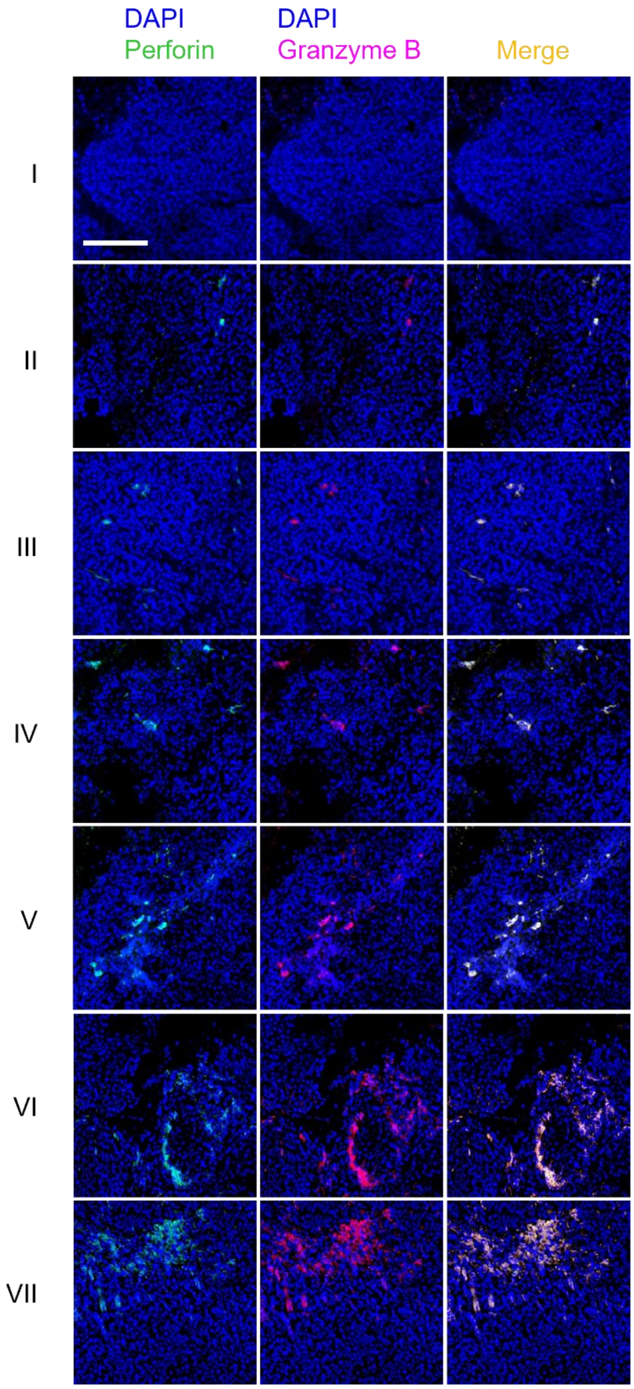

Figure S23. Immunofluorescence images of intratumoral perforin and granzyme B secretion. Scale bar: 200 μm. I, PBS; II, ULNPs@G; III, IL-2F; IV, CLNPs-cRNA^{IL-2F}; V, ULNPs-cRNA^{IL-2F}; VI, CLNPs-cRNA^{IL-2F}@G; VII, ULNPs-cRNA^{IL-2F}@G.

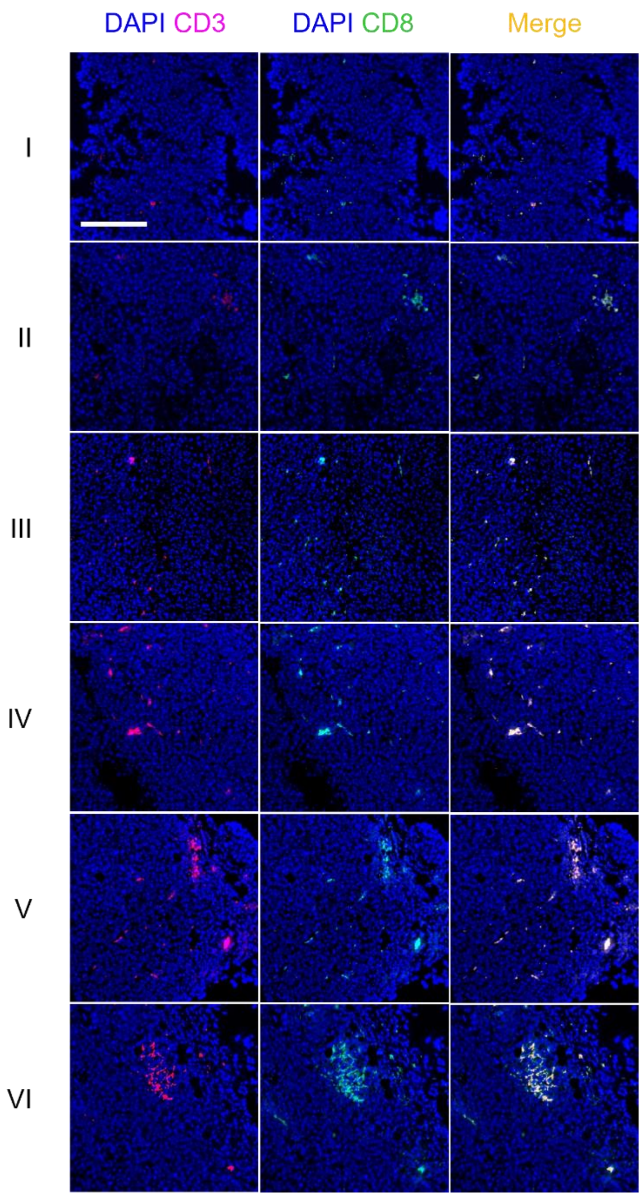

Figure S24. Immunofluorescence images of tumor-infiltrating $CD3^{+}CD8^{+}$ T cells. Scale bar: 200 μm. I, PBS; II, αPD-L1; III, CLNPs-cRNA^{IL-2F}@LG; IV, ULNPs-cRNA^{IL-2F}@LG; V, CLNPs-cRNA^{IL-2F}@LG + αPD-L1; VI, ULNPs-cRNA^{IL-2F}@LG + αPD-L1.

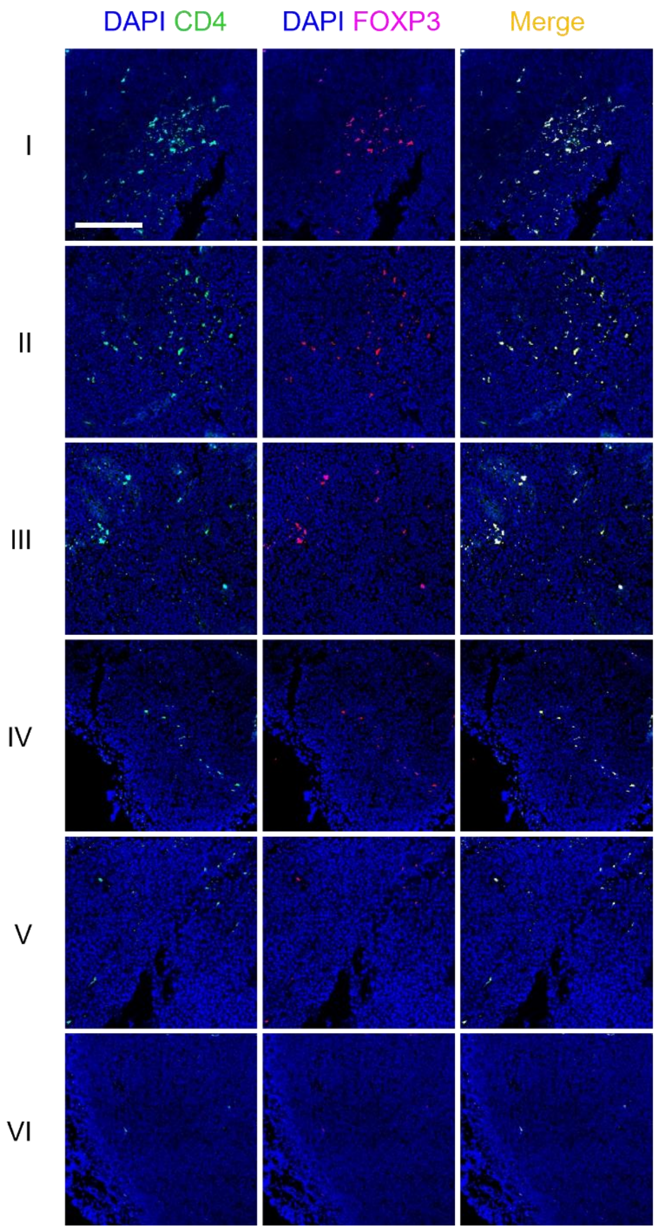

Figure S25. Immunofluorescence images of tumor-infiltrating Treg cells. Scale bar: 200 μm. I, PBS; II, αPD-L1; III, CLNPs-cRNA^{IL-2F}@LG; IV, ULNPs-cRNA^{IL-2F}@LG; V, CLNPs-cRNA^{IL-2F}@LG + αPD-L1; VI, ULNPs-cRNA^{IL-2F}@LG + αPD-L1.

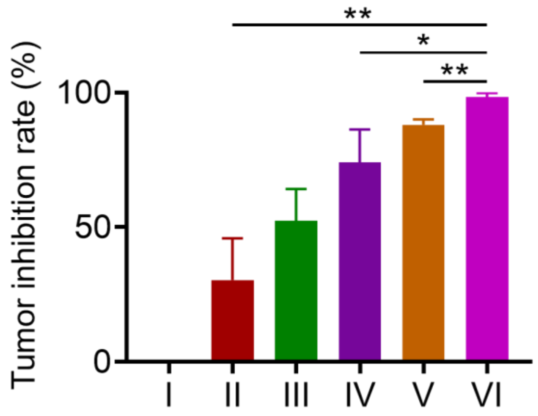

Figure S26. Tumor inhibition rate of the mice after various treatments. I, PBS; II, αPD-L1; III, CLNPs-cRNA^{IL-2F}@LG; IV, ULNPs-cRNA^{IL-2F}@LG; V, CLNPs-cRNA^{IL-2F}@LG + αPD-L1; VI, ULNPs-cRNA^{IL-2F}@LG + αPD-L1. Data represent means ± SD. Statistical differences were calculated using a student's t test (*p < 0.05, **p < 0.01, ***p < 0.001, ****p < 0.0001).

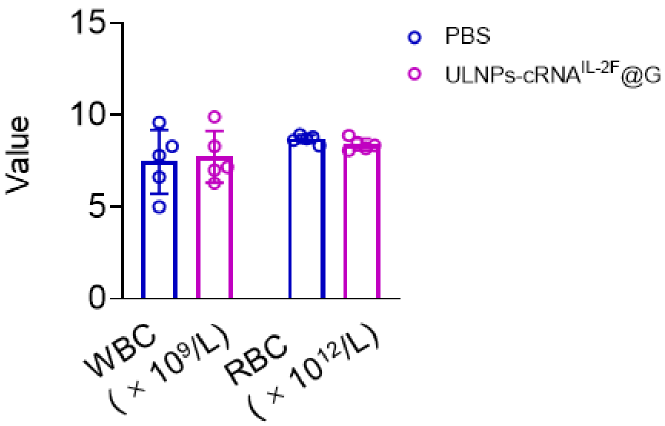

Figure S27. Blood routine tests of the mice after subcutaneous injection of PBS or ULNPs-cRNA^{IL-2F}@G for 24 h.

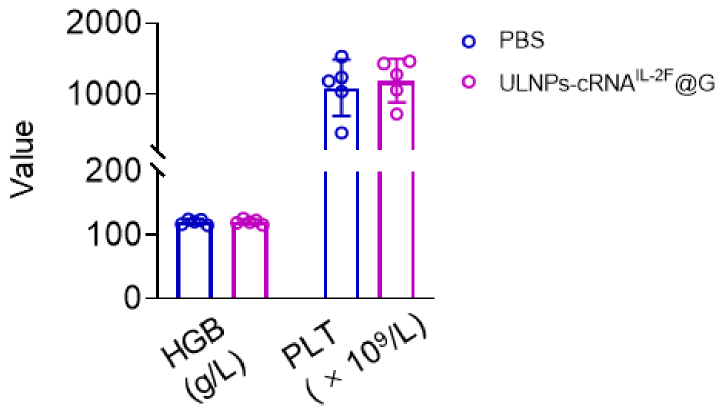

Figure S28. Blood routine tests of the mice after subcutaneous injection of PBS or ULNPs-cRNA^{IL-2F}@G for 24 h.

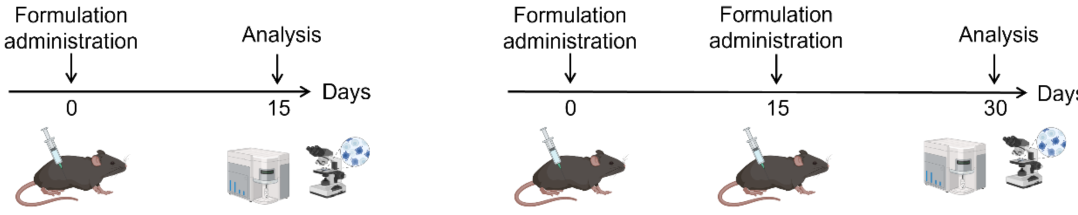

Figure S29. Schematic illustration of safety evaluation on C57BL/6J mice received subcutaneous injection of PBS or ULNPs-cRNA^{IL-2F}@G.

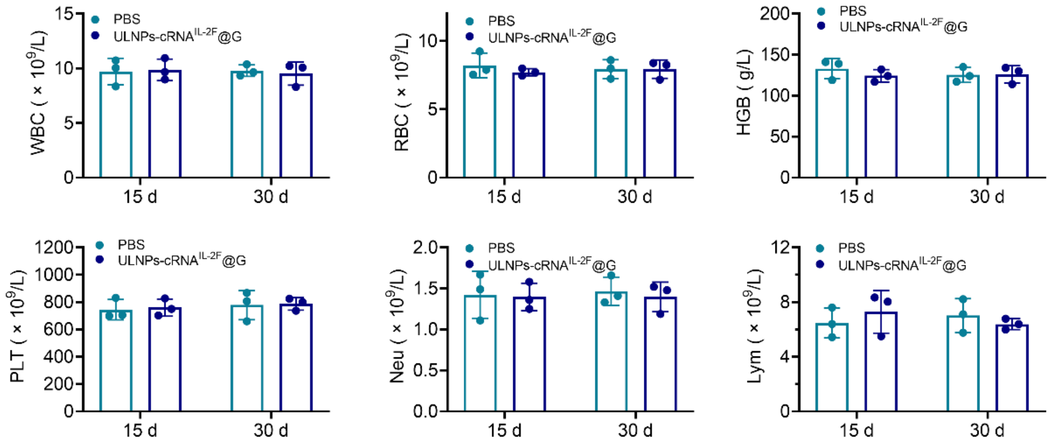

Figure S30. Blood routine test results of mice after various treatments.

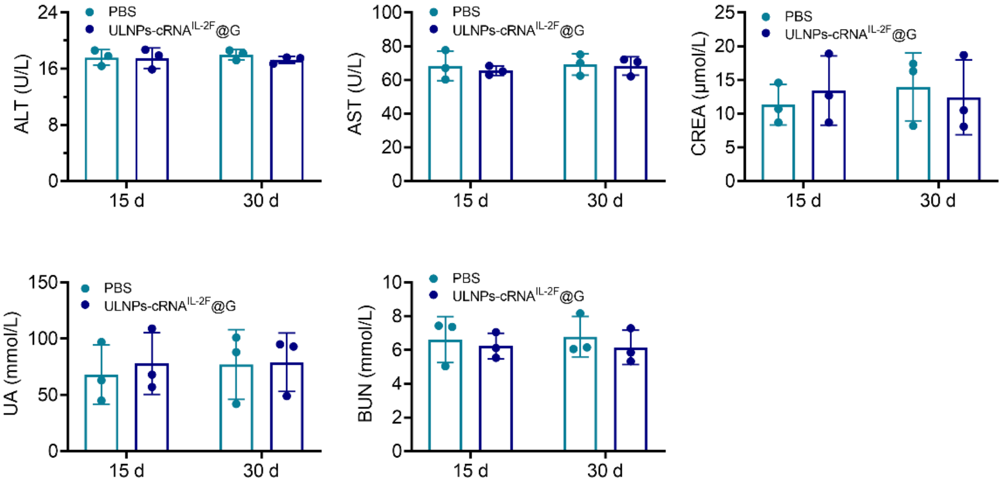

Figure S31. Hepatic and renal function test results of mice after various treatments.

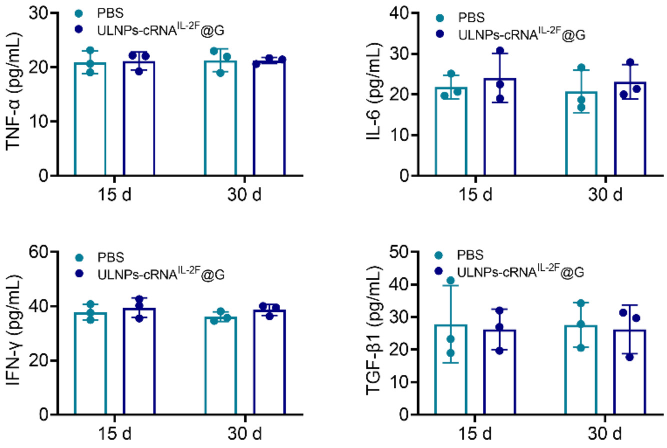

Figure S32. Systemic cytokine results of mice after various treatments.

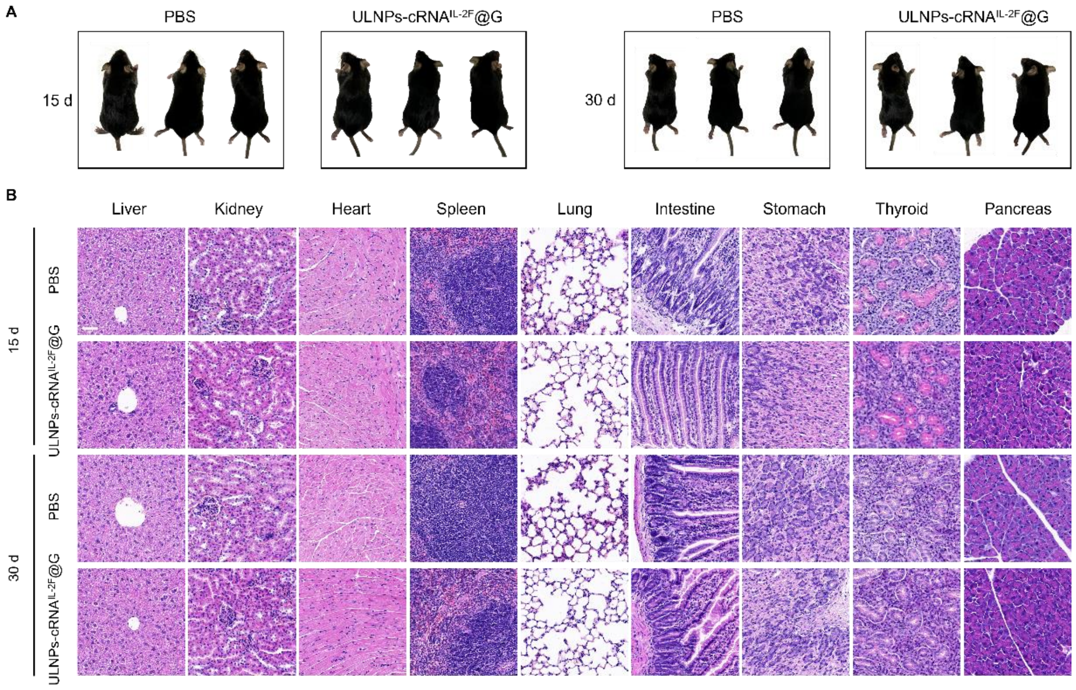

Figure S33. (A) Skin situation of mice after various treatments. (B) H&E staining of organs from mice after various treatments, scale bar: 50 μm.

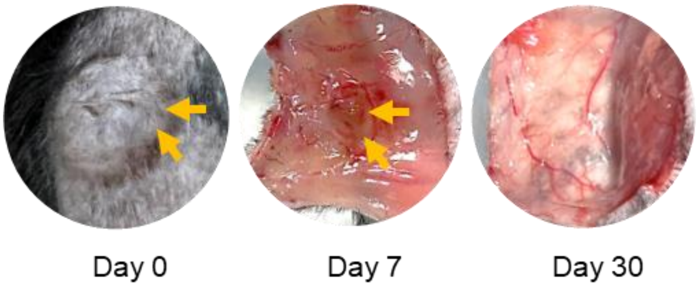

Figure S34. Images of ULNPs-cRNA^{IL-2F}@G after subcutaneous injection at different time points.

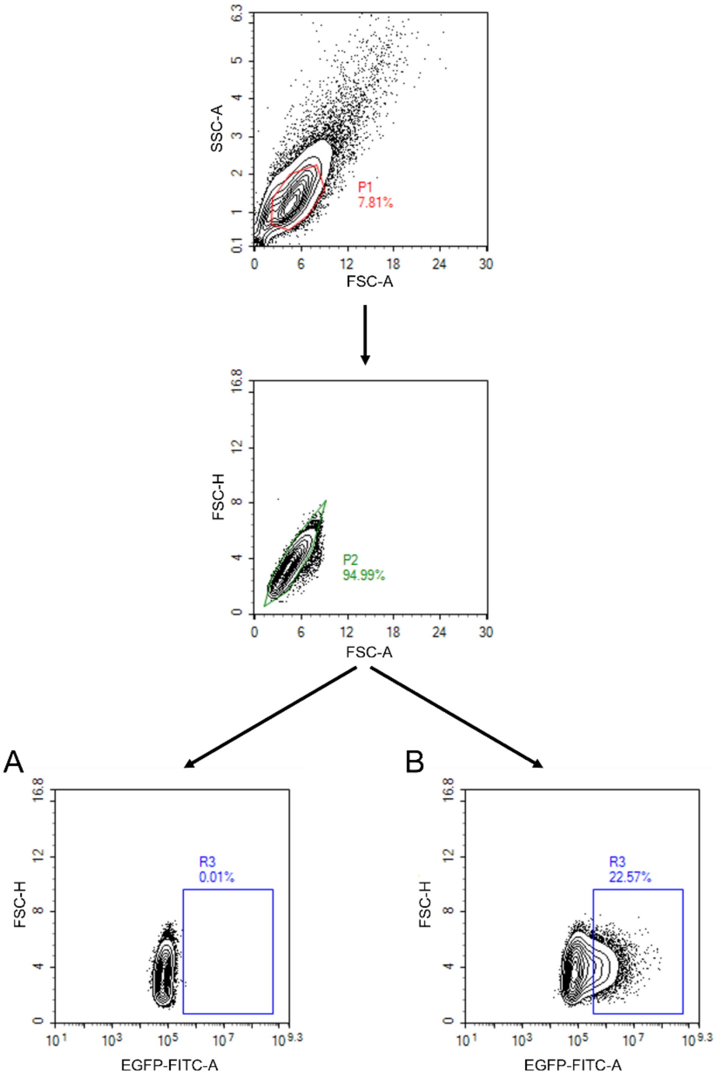

Figure S35. Gating strategy of the flow cytometry data in Figure S11. (**A**) Cells were treated with ULNPs. (**B**) Cells were treated with ULNPs-mRNA^{EGFP}.

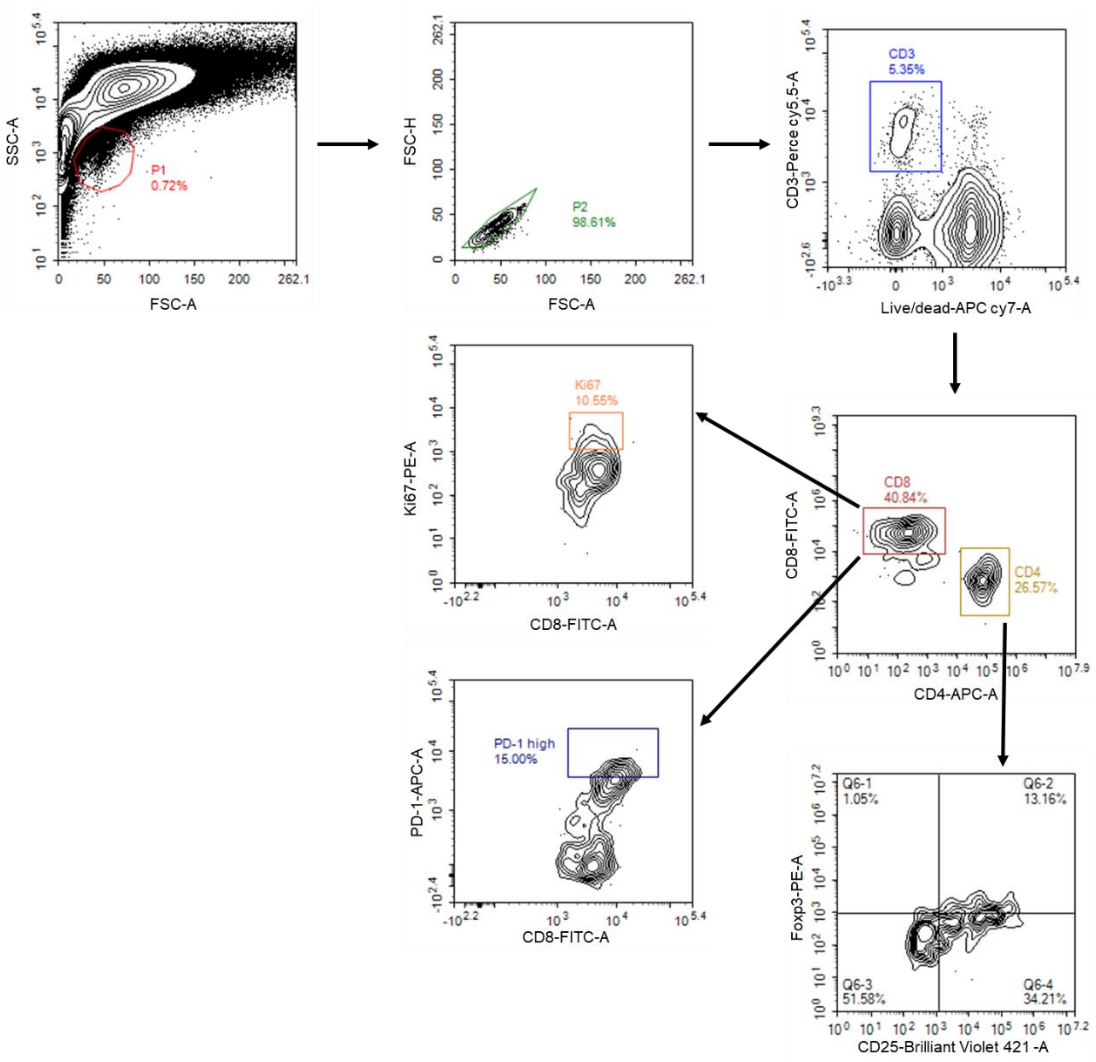

Figure S36. Gating strategy of the flow cytometry data in Figure 4g, Figure 4h, Figure 5g, Figure 4j, and Figure S15.

Supplementary Table 1. Information of antibodies used in this study.

Antibodies	Source	Identifier	Dilution ratio
Fixable viability dye eFluor™ 780	ThermoFisher	65-0865-14	1:5000
Perce-cy5.5 anti-CD3	BioLegend	100218	1:500
FITC anti-CD8a	BioLegend	100706	1:500
APC anti-CD4	BioLegend	100411	1:500
Brilliant Violet 421 anti-CD25	BioLegend	102043	1:500
PE anti-Foxp3	BioLegend	126404	1:500
APC anti-CD279 (PD-1)	BioLegend	135209	1:500
PE anti-Ki67	BioLegend	652404	1:500
TGF-β1	Cell Signaling	3711	1:1000
β-Actin	Abmart	M20011M	1:5000
LC3B	Invitrogen	MA5-37852	1:3000
HRP-conjugated goat anti-mouse	Abmart	M21001S	1:1000
HRP-conjugated goat anti-rabbit	Abmart	M21002S	1:1000
Rhodamine-labeled goat anti-rabbit IgG	Zhongshan Golden Bridge	ZF-0316	1:100
FITC-labeled goat anti-mouse IgG	Zhongshan Golden Bridge	ZF-0312	1:100

Supplementary Table 2. The coding sequence of cRNA^{Luc}, cRNA^{EGFP}, and cRNA$^{IL\text{-}2F}$.

Circular luciferase RNA templete	taatacgactcactataggggagaccctcgaccgtcgattgtccactggtcaacaatagatgacttacaactaat cggaaggtgcagagactcgacgggagctaccctaacgtcaagacgagggtaaagagagagtccaattctcaa agccaataggcagtagcgaaagctgcaagagaatgaaaatccgttgaccttaaacggtcgtgtgggttcaagtc cctccacccccacgccggaaacgcaatagccgaaaaacaaaaaacaaaaaaaacaaaaaaaaaaccaaaaa aacaaaacacaTCTAGATTAAAACAGCCTGTGGGTTGATCCCACCCACAG GCCCATTGGGCGCTAGCACTCTGGTATCACGGTACCTTTGTGCGCCT

	GTTTTATACCCCCTCCCCCAACTGTAACTTAGAAGTAACACACACCG<br>ATCAACAGTCAGCGTGGCACACCAGCCACGTTTTGATCAAGCACTT<br>CTGTTACCCCGGACTGAGTATCAATAGACTGCTCACGCGGTTGAAGG<br>AGAAAGCGTTCGTTATCCGGCCAACTACTTCGAAAAACCTAGTAACA<br>CCGTGGAAGTTGCAGAGTGTTTCGCTCAGCACTACCCCAGTGTAGAT<br>CAGGTCGATGAGTCACCGCATTCCCCACGGGCGACCGTGGCGGTGG<br>CTGCGTTGGCGGCCTGCCCATGGGGAAACCCATGGGACGCTCTAATA<br>CAGACATGGTGCGAAGAGTCTATTGAGCTAGTTGGTAGTCCTCCGGC<br>CCCTGAATGCGGCTAATCCTAACTGCGGAGCACACACCCTCAAGCC<br>AGAGGGCAGTGTGTCGTAACGGGCAACTCTGCAGCGGAACCGACTA<br>CTTTGGGTGTCCGTGTTTCATTTTATTCCTATACTGGCTGCTTATGGTG<br>ACAATTGAGAGATCGTTACCATATAGCTATTGGATTGGCCATCCGGTG<br>ACTAATAGAGCTATTATATATCCCTTTGTTGGGTTTATACCACTTAGCT<br>TGAAAGAGGTTAAAACATTACAATTCATTGTTAAGTTGAATACAGCA<br>AAGCGGCCGCGCCACCATGGAAGACGCCAAAAACATAAAGAAAGG<br>CCCGGCGCCATTCTATCCGCTGGAAGATGGAACCGCTGGAGAGCAA<br>CTGCATAAGGCTATGAAGAGATACGCCCTGGTTCCTGGAACAATTGC<br>TTTTACAGATGCACATATCGAGGTGGACATCACTTACGCTGAGTACTT<br>CGAAATGTCCGTTCGGTTGGCAGAAGCTATGAAACGATATGGGCTGA<br>ATACAAATCACAGAATCGTCGTATGCAGTGAAAACTCTCTTCAATTC<br>TTTATGCCGGTGTTGGGCGCGTTATTTATCGGAGTTGCAGTTGCGCCC<br>GCGAACGACATTTATAATGAACGTGAATTGCTCAACAGTATGGGCAT

	TTCGCAGCCTACCGTGGTGTTCGTTTCCAAAAAGGGGTTGCAAAAA<br>ATTTTGAACGTGCAAAAAAAGCTCCCAATCATCCAAAAAATTATTAT<br>CATGGATTCTAAAACGGATTACCAGGGATTTCAGTCGATGTACACGT<br>TCGTCACATCTCATCTACCTCCCGGTTTTAATGAATACGATTTTGTGC<br>CAGAGTCCTTCGATAGGGACAAGACAATTGCACTGATCATGAACTCC<br>TCTGGATCTACTGGTCTGCCTAAAGGTGTCGCTCTGCCTCATAGAAC<br>TGCCTGCGTGAGATTCTCGCATGCCAGAGATCCTATTTTTGGCAATCA<br>AATCATTCCGGATACTGCGATTTTAAGTGTTGTTCCATTCCATCACGG<br>TTTTGGAATGTTTACTACACTCGGATATTTGATATGTGGATTTCGAGTC<br>GTCTTAATGTATAGATTTGAAGAAGAGCTGTTTCTGAGGAGCCTTCA<br>GGATTACAAGATTCAAAGTGCGCTGCTGGTGCCAACCCTATTCTCCT<br>TCTTCGCCAAAAGCACTCTGATTGACAAATACGATTTATCTAATTTAC<br>ACGAAATTGCTTCTGGTGGCGCTCCCCTCTCTAAGGAAGTCGGGGA<br>AGCGGTTGCCAAGAGGTTCCATCTGCCAGGTATCAGGCAAGGATATG<br>GGCTCACTGAGACTACATCAGCTATTCTGATTACACCCGAGGGGGAT<br>GATAAACCGGGCGCGGTCGGTAAAGTTGTTCCATTTTTTGAAGCGAA<br>GGTTGTGGATCTGGATACCGGGAAAACGCTGGGCGTTAATCAAAGA<br>GGCGAACTGTGTGTGAGAGGTCCTATGATTATGTCCGGTTATGTAAA<br>CAATCCGGAAGCGACCAACGCCTTGATTGACAAGGATGGATGGCTA<br>CATTCTGGAGACATAGCTTACTGGGACGAAGACGAACACTTCTTCAT<br>CGTTGACCGCCTGAAGTCTCTGATTAAGTACAAAGGCTATCAGGTGG<br>CTCCCGCTGAATTGGAATCCATCTTGCTCCAACACCCCAACATCTTC

	GACGCAGGTGTCGCAGGTCTTCCCGACGATGACGCCGGTGAACTTC<br>CCGCCGCCGTTGTTGTTTTGGAGCACGGAAAGACGATGACGGAAAA<br>AGAGATCGTGGATTACGTCGCCAGTCAAGTAACAACCGCGAAAAAG<br>TTGCGCGGAGGAGTTGTGTTTGTGGACGAAGTACCGAAAGGTCTTA<br>CCGGAAAACTCGACGCAAGAAAAATCAGAGAGATCCTCATAAAGGC<br>CAAGAAGGGCGGAAAGATCGCCGTGTGAGTCGACaaaaaacaaaaaacaaaa<br>cggctattatgcgttaccggcgagacgctacggacttaaataattgagccttaaagaagaaattctttaagtggat<br>gctctcaaactcagggaaacctaaatctagttatagacaaggcaatcctgagccaagccgaagtagtaattagta<br>agaccagtggacaatcgacggataacagcatatctag
Circular<br>EGFP<br>RNA<br>templete	TCGAGCATCGTAATACGACTCACTATAGGGCGAATTGGGAGACCCTC<br>GACCGTCGATTGTCCACTGGTCAACAATAGATGACTTACAACTAATC<br>GGAAGGTGCAGAGACTCGACGGGAGCTACCCTAACGTCAAGACGA<br>GGGTAAAGAGAGAGTCCAATTCTCAAAGCCAATAGGCAGTAGCGAA<br>AGCTGCAAGAGAATGAAAATCCGTTGACCTTAAACGGTCGTGTGGG<br>TTCAAGTCCCTCCACCCCCACGCCGGAAACGCAATAGCCGAAAAAC<br>AAAAAACAAAAAAAACAAAAAAAAAACCAAAAAAACAAAACACA<br>TTAAAACAGCCTGTGGGTTGATCCCACCCACAGGCCCATTGGGCGCT<br>AGCACTCTGGTATCACGGTACCTTTGTGCGCCTGTTTTATACCCCCTC<br>CCCCAACTGTAACTTAGAAGTAACACACACCGATCAACAGTCAGCG<br>TGGCACACCAGCCACGTTTTGATCAAGCACTTCTGTTACCCCGGACT<br>GAGTATCAATAGACTGCTCACGCGGTTGAAGGAGAAAGCGTTCGTT<br>ATCCGGCCAACTACTTCGAAAAACCTAGTAACACCGTGGAAGTTGC

	AGAGTGTTTCGCTCAGCACTACCCCAGTGTAGATCAGGTCGATGAGT<br>CACCGCATTCCCCACGGGCGACCGTGGCGGTGGCTGCGTTGGCGGC<br>CTGCCCATGGGGAAACCCATGGGACGCTCTAATACAGACATGGTGCG<br>AAGAGTCTATTGAGCTAGTTGGTAGTCCTCCGGCCCCTGAATGCGGC<br>TAATCCTAACTGCGGAGCACACACCCTCAAGCCAGAGGGCAGTGTG<br>TCGTAACGGGCAACTCTGCAGCGGAACCGACTACTTTGGGTGTCCG<br>TGTTTCATTTTATTCCTATACTGGCTGCTTATGGTGACAATTGAGAGAT<br>CGTTACCATATAGCTATTGGATTGGCCATCCGGTGACTAATAGAGCTA<br>TTATATATCCCTTTGTTGGGTTTATACCACTTAGCTTGAAAGAGGTTA<br>AAACATTACAATTCATTGTTAAGTTGAATACAGCAAAGATATCGCCAC<br>CATGGTGAGCAAGGGCGAGGAGCTGTTCACCGGGGTGGTGCCCATC<br>CTGGTCGAGCTGGACGGCGACGTAAACGGCCACAAGTTCAGCGTGT<br>CCGGCGAGGGCGAGGGCGATGCCACCTACGGCAAGCTGACCCTGA<br>AGTTCATCTGCACCACCGGCAAGCTGCCCGTGCCCTGGCCCACCCTC<br>GTGACCACCCTGACCTACGGCGTGCAGTGCTTCAGCCGCTACCCCG<br>ACCACATGAAGCAGCACGACTTCTTCAAGTCCGCCATGCCCGAAGG<br>CTACGTCCAGGAGCGCACCATCTTCTTCAAGGACGACGGCAACTAC<br>AAGACCCGCGCCGAGGTGAAGTTCGAGGGCGACACCCTGGTGAAC<br>CGCATCGAGCTGAAGGGCATCGACTTCAAGGAGGACGGCAACATCC<br>TGGGGCACAAGCTGGAGTACAACTACAACAGCCACAACGTCTATAT<br>CATGGCCGACAAGCAGAAGAACGGCATCAAGGTGAACTTCAAGATC<br>CGCCACAACATCGAGGACGGCAGCGTGCAGCTCGCCGACCACTACC

	AGCAGAACACCCCCATCGGCGACGGCCCCGTGCTGCTGCCCGACAA<br>CCACTACCTGAGCACCCAGTCCGCCCTGAGCAAAGACCCCAACGAG<br>AAGCGCGATCACATGGTCCTGCTGGAGTTCGTGACCGCCGCCGGGA<br>TCACTCTCGGCATGGACGAGCTGTACAAGTAAGATATCAAAAAACA<br>AAAAACAAAACGGCTATTATGCGTTACCGGCGAGACGCTACGGACT<br>TAAATAATTGAGCCTTAAAGAAGAAATTCTTTAAGTGGATGCTCTCA<br>AACTCAGGGAAACCTAAATCTAGTTATAGACAAGGCAATCCTGAGCC<br>AAGCCGAAGTAGTAATTAGTAAGACCAGTGGACAATCGACGGATAA<br>CAGCATATCTAG
Circular<br>IL-2F<br>RNA<br>templete	TCGAGCATCGTAATACGACTCACTATAGGGCGAATTGGGAGACCCTC<br>GACCGTCGATTGTCCACTGGTCAACAATAGATGACTTACAACTAATC<br>GGAAGGTGCAGAGACTCGACGGGAGCTACCCTAACGTCAAGACGA<br>GGGTAAAGAGAGAGTCCAATTCTCAAAGCCAATAGGCAGTAGCGAA<br>AGCTGCAAGAGAATGAAAATCCGTTGACCTTAAACGGTCGTGTGGG<br>TTCAAGTCCCTCCACCCCCACGCCGGAAACGCAATAGCCGAAAAAC<br>AAAAAACAAAAAAAACAAAAAAAAAACCAAAAAAACAAAACACA<br>TTAAAACAGCCTGTGGGTTGATCCCACCCACAGGCCCATTGGGCGCT<br>AGCACTCTGGTATCACGGTACCTTTGTGCGCCTGTTTTATACCCCCTC<br>CCCCAACTGTAACTTAGAAGTAACACACACCGATCAACAGTCAGCG<br>TGGCACACCAGCCACGTTTTGATCAAGCACTTCTGTTACCCCGGACT<br>GAGTATCAATAGACTGCTCACGCGGTTGAAGGAGAAAGCGTTCGTT<br>ATCCGGCCAACTACTTCGAAAAACCTAGTAACACCGTGGAAGTTGC

	AGAGTGTTTCGCTCAGCACTACCCCAGTGTAGATCAGGTCGATGAGT<br>CACCGCATTCCCCACGGGCGACCGTGGCGGTGGCTGCGTTGGCGGC<br>CTGCCCATGGGGAAACCCATGGGACGCTCTAATACAGACATGGTGCG<br>AAGAGTCTATTGAGCTAGTTGGTAGTCCTCCGGCCCCTGAATGCGGC<br>TAATCCTAACTGCGGAGCACACACCCTCAAGCCAGAGGGCAGTGTG<br>TCGTAACGGGCAACTCTGCAGCGGAACCGACTACTTTGGGTGTCCG<br>TGTTTCATTTTATTCCTATACTGGCTGCTTATGGTGACAATTGAGAGAT<br>CGTTACCATATAGCTATTGGATTGGCCATCCGGTGACTAATAGAGCTA<br>TTATATATCCCTTTGTTGGGTTTATACCACTTAGCTTGAAAGAGGTTA<br>AAACATTACAATTCATTGTTAAGTTGAATACAGCAAAGATATCGCCAC<br>CATGGAGACAGACACACTCCTGCTATGGGTACTGCTGCTCTGGGTTC<br>CAGGTTCCACTGGTGACTCGCGACGTACGGCCCCTACAAGCAGCAG<br>CACCAAGAAGACCCAGCTGCAGCTGGAACACCTGCTGCTGGATCTG<br>CAGATGATCCTGAACGGCATCAACAACTACAAGAACCCCAAGCTGA<br>CCCGGATGCTGACCTTCAAGTTCTACATGCCCAAGAAGGCCACCGA<br>GCTGAAGCACCTCCAGTGTCTGGAGGAGGAGCTGAAGCCTCTGGAG<br>GAAGTGCTGAACCTGGCCCAGAGCAAGAACTTCCACTTAAGACCCA<br>GGGACTTAATCTCCAACATCAACGTGATAGTGCTGGAACTGAAGGG<br>CAGCGAGACCACCTTCATGTGCGAGTACGCCGACGAGACCGCTACC<br>ATCGTGGAGTTCCTGAACCGCTGGATCACCTTTTGCCAGAGCATCAT<br>CAGCACACTGACCTTCGAAGAGCCCAAGAGCTGCGACAAGACCCA<br>CACCTGTCCCCCTTGTCCTGCCCCTGAGCTGCTGGGCGGACCCAGC

	GTGTTCCTGTTCCCCCCAAAGCCCAAGGACACCCTGATGATCAGCCG<br>GACCCCCGAAGTGACCTGCGTGGTGGTGGACGTGTCCCACGAGGAC<br>CCTGAAGTGAAGTTCAATTGGTACGTGGACGGCGTGGAGGTGCACA<br>ACGCCAAGACCAAGCCCCGGGAGGAACAGTACAACAGCACCTACC<br>GGGTGGTGTCCGTGCTGACCGTGCTGCACCAGGACTGGCTGAACGG<br>CAAAGAGTACAAGTGCAAGGTCTCCAACAAGGCCCTGCCTGCCCCC<br>ATCGAAAAGACCATCAGCAAGGCCAAGGGCCAGCCCAGAGAACCC<br>CAGGTGTACACCCTGCCCCCCAGCAGAGATGAGCTGACCAAGAACC<br>AGGTGTCCCTGACCTGCCTGGTCAAGGGCTTCTACCCCAGCGATATC<br>GCCGTGGAGTGGGAGAGCAACGGCCAGCCTGAGAACAACTACAAG<br>ACCACCCCCCCTGTGCTGGACAGCGATGGCAGCTTCCTCTACAGCA<br>AACTGACCGTGGACAAGAGCCGGTGGCAGCAGGGCAACGTGTTCA<br>GCTGCAGCGTGATGCACGAGGCCCTGCACAACCACTACACCCAGAA<br>GTCCCTGAGCCTGAGCCCCGGCAAATAGGATATCAAAAAACAAAAA<br>ACAAAACGGCTATTATGCGTTACCGGCGAGACGCTACGGACTTAAAT<br>AATTGAGCCTTAAAGAAGAAATTCTTTAAGTGGATGCTCTCAAACTC<br>AGGGAAACCTAAATCTAGTTATAGACAAGGCAATCCTGAGCCAAGC<br>CGAAGTAGTAATTAGTAAGACCAGTGGACAATCGACGGATAACAGC<br>ATATCTAG
